# Development of novel FP-based probes for live-cell imaging of nitric oxide dynamics

**DOI:** 10.1038/ncomms10623

**Published:** 2016-02-04

**Authors:** Emrah Eroglu, Benjamin Gottschalk, Suphachai Charoensin, Sandra Blass, Helmut Bischof, Rene Rost, Corina T. Madreiter-Sokolowski, Brigitte Pelzmann, Eva Bernhart, Wolfgang Sattler, Seth Hallström, Tadeusz Malinski, Markus Waldeck-Weiermair, Wolfgang F. Graier, Roland Malli

**Affiliations:** 1 Institute of Molecular Biology and Biochemistry, Center of Molecular Medicine, Medical University of Graz, Harrachgasse 21/III, 8010 Graz, Austria; 2 Institute of Biophysics, Center of Physiological Medicine, Medical University of Graz, Harrachgasse 21/IV, 8010 Graz, Austria; 3 Institute of Physiological Chemistry, Center of Physiological Medicine, Medical University of Graz, Harrachgasse 21/II, 8010 Graz, Austria; 4 Nanomedical Research Laboratory, Department of Chemistry and Biochemistry, Ohio University, 350 West State Street, Athens, Ohio 45701, USA

## Abstract

Nitric oxide (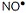) is a free radical with a wide range of biological effects, but practically impossible to visualize in single cells. Here we report the development of novel multicoloured fluorescent quenching-based 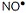 probes by fusing a bacteria-derived 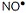-binding domain close to distinct fluorescent protein variants. These genetically encoded 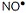 probes, referred to as geNOps, provide a selective, specific and real-time read-out of cellular 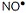 dynamics and, hence, open a new era of 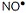 bioimaging. The combination of geNOps with a Ca^2+^ sensor allowed us to visualize 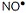 and Ca^2+^ signals simultaneously in single endothelial cells. Moreover, targeting of the 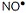 probes was used to detect 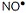 signals within mitochondria. The geNOps are useful new tools to further investigate and understand the complex patterns of 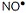 signalling on the single (sub)cellular level.

## Abstract (web summary)

Nitric oxide is a volatile free radical second messenger with a large number of biological effects. Here Eroglu *et al*. develop genetically encoded fluorescent biosensors for nitric oxide and use them to visualise subcellular nitric oxide dynamics in single cells.

## Graphical abstract

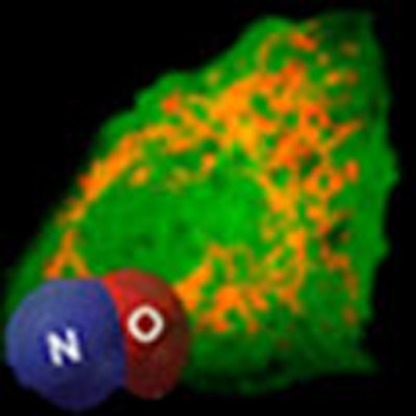

The nitric oxide radical (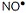) is one of the most studied molecule^1^. The interest in 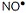 is based on the important roles this radical plays in the chemical industry, in environmental ecology and, above all, in biology, where it represents one of the most versatile mediators in the (cardio-)vascular, nervous and immune systems^2^. Recent studies indicate that 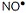 is also a crucial messenger in tumour cell signalling^3^, plant–microbe interactions^4^ and the development of resistance of bacteria against antibiotics^5^. The wide range of physiological and pathological effects of 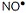 are partially induced by the reactivity of the molecule, which is able to modify biomolecules including proteins, lipids and nucleic acids^6^. In addition, 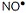 works as a signalling molecule via binding to metalloproteins with specific iron(II) or zinc(II)-containing 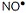-binding domains. In these domains, 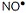 reversibly interacts with the metal ion and thereby modulates the conformation and activity of the whole signalling protein^7^. Although the fundamental roles of 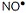 in biology have been established undoubtedly, many questions remain unanswered, because of limitations of the methods available to detect 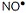 in biological samples^8^. Multiple methods to determine 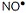 concentrations including organ assays^9^, cell assays^10^, enzymatic assays^11^, electrochemical microelectrodes^12^, spectroscopic measurements^13^ and fluorescent probes^14^,^15^ have been developed. However, despite the availability of such a broad range of 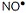 detection techniques, research activities designed to investigate the complex metabolism and signalling patterns of 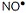 in physiology and pathology suffer from the lack of practicable methods for intracellular, single-cell 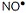 detection^8^. To overcome this limitation, we aimed to develop genetically encoded fluorescent probes that specifically and directly respond to 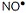, thus providing a quantifiable and real-time readout of cellular 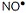 dynamics. Therefore, we designed, produced and characterized various genetically encoded 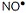 probes (geNOps) by selecting a suitable 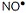-binding domain that was conjugated with differently coloured fluorescent protein (FP) variants. We assumed that specific 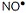 binding close to FP in such constructs considerably influences the fluorescence signal by affecting the electron density within certain amino acids forming the chromophore. In this study, we demonstrate that such fluorescent chimeras, referred to as geNOps, represent a completely novel class of 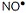 indicators that allow direct imaging of (sub)cellular 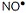 dynamics in real time.

## Results

### Generation of differently coloured geNOps

**Figure 1:**
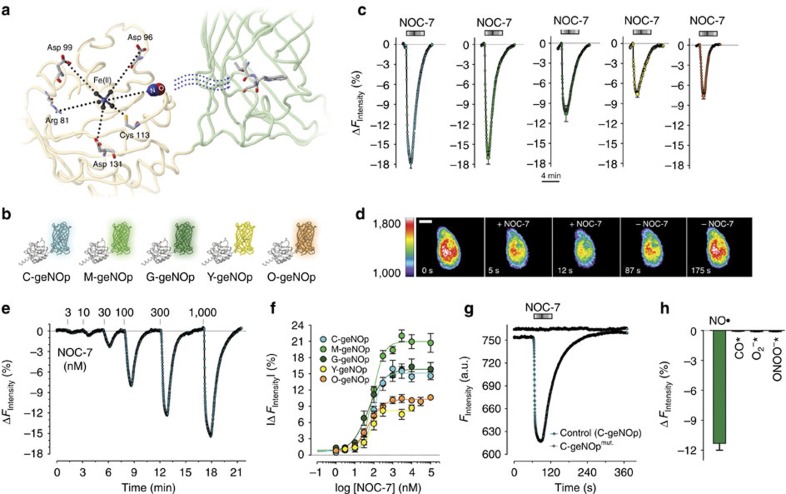
Fusion of the bacterial 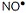-binding GAF domain to fluorescent proteins, resulting in differently coloured fluorescent quenching-based 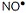 probes, the geNOps. (**a**) Predicted three-dimensional structure of geNOps. (**b**) Schematic overview of differently coloured geNOps. (**c**) Average curves (mean±s.e.m.) over time of normalized delta fluorescence signals in % of the differently coloured geNOps signals in response to 10 μM NOC-7 (*n*=10 for C-geNOp cyan curve; *n*=12 for M-geNOp light green curve; *n*=11 for G-geNOp dark-green curve; *n*=13 for Y-geNOp yellow curve; *n*=9 for O-geNOp orange curve). Experiments were performed using HeLa cells. (**d**) Representative pseudo-coloured images of a HeLa cell expressing O-geNOp before cell treatment (0 s), upon addition of 10 μM NOC-7 (5 and 12 s) and upon the removal of NOC-7 (87 and 175 s). Scale bar, 10 μm. See also Supplementary Video 1. (**e**) Fluorescence intensity change in % versus time of a single HeLa cell expressing C-geNOp in response to different concentrations of NOC-7. (**f**) Concentration response curves showing the effects of different NOC-7 concentrations on fluorescence intensities of the differently coloured geNOps that were expressed in HeLa cells. Points represent average values±s.e.m.; *n*=5 for C-geNOp, *n*=8-11 for M-geNOp; *n*=5–6 for G-geNOp; *n*=3 for Y-geNOp; *n*=3–5 for O-geNOp. (**g**) Representative curves showing fluorescence over time of wild-type C-geNOp and C-geNOp^mut^ upon addition of 10 μM NOC-7 to HeLa cells. Statistics are shown in Supplementary Fig. 7. (**h**) Bars representing maximal delta fluorescence signals±s.e.m. of G-geNOp expressed in HeLa cells in response to 10 μM NOC-7 (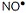, green column, *n*=26), 100 μM of the CO-releasing compound CORM-3 (CO, *n*=16), 100 μM KO_2_ (O_2_, *n*=12) or 100 μM peroxynitrite (ONOO^−^, *n*=7). *P<0.05 versus control using the unpaired *t*-test.

Out of a limited number of known 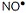-binding domains, we selected the GAF domain of the enhancer-binding protein NorR, a transcription factor of the enteric bacterium *Escherichia coli*^16^,^17^, for the development of fluorescent geNOps. Being bacteria-derived, the GAF domain of NorR was assumed not to interfere with signalling pathways in higher cells. In addition, the bacterial GAF domain is a small, simply built and specific 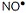-binding domain with a non-haem iron(II) centre^17^, which appears suitable for bringing the 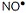 radical in close vicinity to the chromophore of a conjugated FP. Computational calculation of the three-dimensional structure of a chimeric construct, which consists of a single FP fused to the N terminus of the GAF domain, predicted that 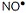 binds close to the FP chromophore and might thereby affect fluorescence (Fig. 1a). On the basis of this computation, we produced five different geNOps with different approved FP variants covering a broad colour range (Fig. 1b). To test whether 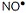 binding to such chimeras affects the fluorescence of the conjugated FPs dependently or independently from their structure and origin, the used FP variants were either mutated versions of the *Aequorea*-derived wild-type green fluorescent protein (GFP; super enhanced cyan fluorescent protein (ECFP)^18^ in cyan geNOp (C-geNOp) and enhanced GFP (EGFP)^19^ in green geNOp (G-geNOp)) or circularly permuted FP variants (GEM^20^ in mint green geNOp (M-geNOp) and circularly permuted Venus^19^ in yellow geNOp (Y-geNOp)) or a *Coral*-derived FP (monomer Kusabira orange mKO_k_ (ref. 21) in orange geNOp (O-geNOp)) (Fig. 1b).

### Characterization of geNOps in living cells

The impact of 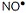 on the fluorescence intensity of the different FP variants within geNOps was examined in HeLa cells expressing these differently coloured chimeras. As expected, expression rates of geNOps in HeLa cells were comparable to those of other genetically encoded probes and FPs alone (Supplementary Fig. 1), demonstrating that the novel protein-based 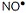 probes are not cytotoxic. To supply the GAF domain of the expressed constructs with sufficient iron(II) (Fe^2+^) required for 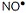 binding^16^,^22^, HeLa cells were incubated in a medium containing Fe^2+^ fumarate and vitamin C for 10 min before fluorescence microscopy. This procedure did not affect the morphology, viability and metabolic activity of different cell types (Supplementary Fig. 2), indicating that the usability of geNOps is not limited by iron(II) supplementation. Addition of NOC-7, a potent 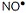 donor^15^ via a perfusion system to the microscope bath, instantly reduced the fluorescence intensity of all the differently coloured geNOps by 7–18% with a high signal-to-noise ratio (Fig. 1c; Supplementary Video 1). A strong linear correlation between the basal fluorescence and the 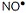-induced quenching effect was observed over a large range of fluorescence intensity (Supplementary Fig. 3). This is an important feature of the non-ratiometric probes for simple absolute quantification of cellular 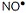 concentrations by normalization (Supplementary Note 1). Removal of NOC-7 completely restored fluorescence, demonstrating the full reversibility of the quenching effect of 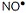 on the different FP variants in the responding chimeras (Fig. 1c–e,g; Supplementary Video 1). These results proved that fusion of the bacterial 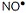-binding GAF domain to FP variants results in C-geNOp, M-geNOp, G-geNOp, Y-geNOp and O-geNOp (Fig. 1b), allowing imaging of cellular 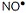 dynamics in real time and in a multichromatic manner. Experiments using sodium nitroprusside (SNP), another 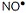-producing compound in cells^23^, showed homogenous signals in response to a number of consecutive 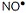 donor pulses (Supplementary Fig. 4), indicating that geNOps are highly stable sensors that enable the recording of extensive 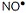 fluctuations over long time. The consecutive addition and removal of different concentrations of NOC-7 (1–100 μM) revealed that the differently coloured geNOps respond in a concentration-dependent manner (Fig. 1e,f) with similar sensitivities (Fig. 1f). The effector concentration for half-maximum response of NOC-7 to induce fluorescence quenching of geNOps was found to be between 50 and 94 nM (Fig. 1f; Supplementary Table 1). Considering the short half-time of NOC-7 (ref. 24) and 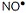 (ref. 25), these results indicate that geNOps are suitable to recording cellular 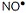 concentrations in the low physiological nM range. However, oxidation of Fe^2+^ to Fe^3+^ by hydrogen peroxide (H_2_O_2_) (Supplementary Fig. 5) or a suboptimal supply of geNOps with Fe^2+^ (Supplementary Fig. 6) significantly reduced the response to the 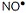 donor. These findings support the idea that nitrosylation of Fe^2+^ of the non-haem iron(II) centre within the GAF domain is essential to induce fluorescence quenching of the attached FP. We further confirmed the Fe^2+^-dependent 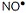-sensing mechanism of geNOps by generating a mutant lacking the arginines at position 75 (deletion) and 81 (R81G), which are essential for the coordinative binding of Fe^2+^ in the non-haem iron(II) centre^16^,^17^ (Supplementary Fig. 7). In contrast to functional geNOps, the fluorescence signal of this mutated construct remained unaffected by the addition of high concentrations of the 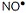 donor to cells expressing the mutated probe (Fig. 1g). In line with these findings, increasing the 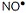 concentration in cells expressing the same FP variants alone or fused to either Ca^2+^- or ATP-binding domains did not impact any of these fluorescence signals (Supplementary Fig. 8). This indicates that the 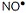 radical, even at high concentrations, does not directly affect the fluorescence of FPs. Consistent with this assumption, the addition of NOC-7 did not affect the fluorescence of HyPer, a genetically encoded H_2_O_2_ probe^26^, which showed a clear reduction of fluorescence upon cell treatment with 50 μM H_2_O_2_ (Supplementary Fig. 9). Contrariwise, the fluorescence of C-geNOp was considerably quenched by adding NOC-7 but remained unaffected by administration of H_2_O_2_, showing that geNOps do not respond to cellular H_2_O_2_ fluctuations (Supplementary Fig. 9). To further examine the selectivity of geNOps, compounds chemically related to 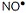, including carbon monoxide, superoxide and peroxynitrite, were tested. While the used compounds have been shown to at least partially diffuse across the plasma membrane of cells^27^,^28^,^29^, none of these compounds affected the geNOp fluorescence signal in HeLa cells, demonstrating the high selectivity of the sensor in its exclusive response to intracellular 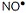 fluctuations (Fig. 1h). As superoxide anions as well as peroxynitrite might not fully penetrate into cells, we also generated a glycosylphosphatidylinositol (GPI)-anchored C-geNOp (GPI-C-geNOp), which localized at the outer surface of the cell membrane (Supplementary Fig. 10a,b). GPI-C-geNOp strongly responded to the addition of 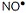 donors (Supplementary Fig. 10c), indicating that the probe remains functional upon targeting to the outer surface of the plasma membrane. Addition of neither superoxide anions nor peroxynitrite significantly affected the fluorescence of GPI-C-geNOp (Supplementary Fig. 10c), confirming the high 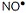 selectivity of geNOps. Moreover, the responsiveness of geNOps to 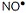 remained at different intracellular pH values (Supplementary Fig. 11). Due to the general pH sensitivity of FPs^19^, the fluorescence of geNOps was altered upon changes of the intracellular proton concentration (Supplementary Fig. 12). O-geNOp containing mKO_k_ (ref. 21) showed the highest pH stability between pH 7 and 9 (Supplementary Fig. 12). Expectedly, the pH-dependent effects on the fluorescence intensity of functional C-geNOp and G-geNOp were equal to that of respective 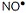-insensitive mutated constructs (Supplementary Fig. 13). Thus, we assume that a clear discrimination between real cellular 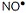 and pH fluctuations is possible by comparing measurements using on the one hand functional 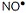 probes and on the other hand mutated geNOps (geNOp^mut^) under the same experimental conditions.

### Generation of mitochondria-targeted geNOps

**Figure 2:**
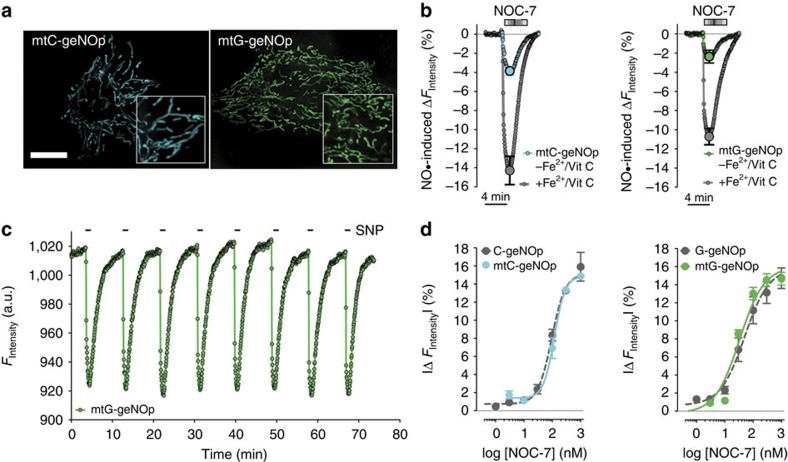
The properties of geNOps remain unaffected upon mitochondria targeting. (**a**) Confocal images of HeLa cells expressing either mtC-geNOp (left image) or mtG-geNOp (right image). Scale bar, 10 μm. (**b**) Normalized average curves±s.e.m. of mtC-geNOp (left panel) and mtG-geNOp (right panel) signals with (*n*=4 for mtC-geNOp; *n*=7 for mtG-geNOp) and without (*n*=5 for mtC-geNOp; *n*=4 for mtG-geNOp) iron(II)/vitamin C pretreatment. Experiments were performed using HeLa cells. (**c**) Representative original curve showing fluorescence over time of mtG-geNOp expressed in HeLa cells in response to consecutive applications of 3 mM SNP (*n*=3). (**d**) Concentration response curves showing the effects of different NOC-7 concentrations on fluorescence intensities of either mtC-geNOp (left panel, cyan curve, *n*=4) versus C-geNOp (left panel, grey curve, for *n* see Fig. 1f) or mtG-geNOp (right panel, green curve, *n*=6) versus G-geNOp (right panel grey curve, for *n* see Fig. 1f). Experiments were performed using HeLa cells. Points represent average values±s.e.m.

Several studies point to a particular role of 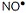 within mitochondria^30^. However, real-time detection of 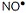 signals within mitochondria in intact cells has not been accomplished so far. Accordingly, we tested whether mitochondria-targeted geNOps (mt-geNOps) allow to overcome this limitation. For this purpose, we constructed mtC-geNOp and mtG-geNOp by fusing a mitochondria-targeting sequence to the N terminus of respective probes. Expression of mtC-geNOp and mtG-geNOp showed clear organelle localization of the constructs (Fig. 2a). Both mtC-geNOp and mtG-geNOp co-localized with MitoTrackerRed, confirming correct targeting of the 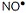 probes to mitochondria (Supplementary Fig. 14). To test the functionality of mitochondria-targeted geNOps, cells expressing these probes were treated with NOC-7. Similar to the non-targeted probes, addition of the 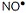 donor instantly and significantly reduced the fluorescence intensity of mtC-geNOp and mtG-geNOp (Fig. 2b), demonstrating the efficiency of mitochondria-targeted geNOps. The 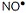-induced quenching of the fluorescence of mt-geNOps was again boosted by Fe^2+^ supplementation (Fig. 2b). Mitochondria targeting did not affect the quality of geNOps to detect consecutive pulses of 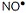 over a long period of time (Fig. 2c). In addition, both mtC-geNOp and mtG-geNOp showed similar sensitivities and responsiveness to different concentrations of NOC-7 compared with the respective non-targeted 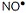 probes (Fig. 2d). These data prove that mitochondria-targeted geNOps can be used for live-cell imaging of 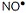 signals within these cellular organelles.

### Imaging of cellular 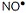 signals in response to 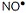 donors

**Figure 3:**
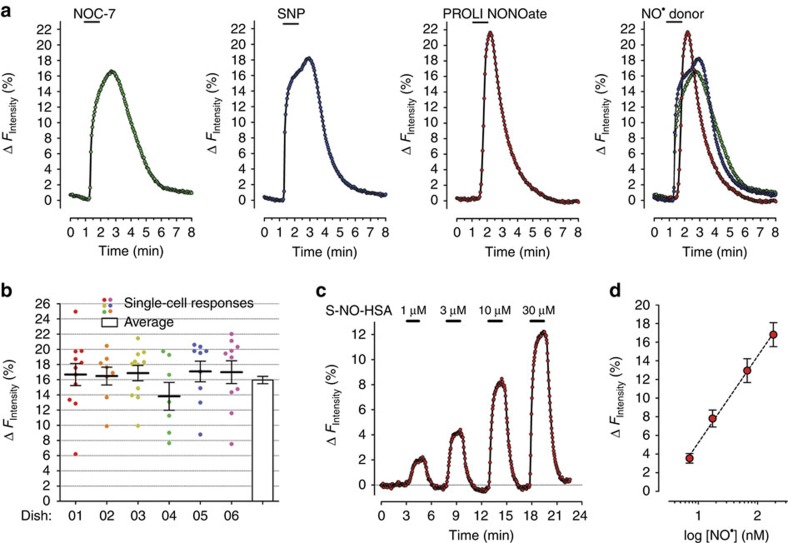
Imaging of cellular 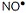 dynamics with geNOps in response to different 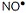-liberating molecules. (**a**) Representative single HeLa cell 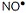 dynamics in response to 1 μM NOC-7, 1 mM SNP or 1 μM PROLI NONOate. Cells expressing C-geNOp were imaged. Inverted curves (1−*F*/*F*0 in %) are shown. Average curves with s.e.m. are shown in Supplementary Fig. 15. (**b**) Scatter dot plot showing maximal single-cell C-geNOp signals in response to 10 μM NOC-7 on different dishes. White column represents the normalized average±s.e.m. C-geNOp signal of all single HeLa cells (*n*=67). (**c**) Intracellular 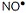 dynamics of a single HeLa cell expressing C-geNOp in response to different concentrations of S-NO-HSA (curve is inverted). (**d**) Respective Δ*F*_Intensity_ mean values±s.e.m. are blotted against 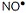 concentrations that are released by 1, 3, 10 and 30 μM S-NO-HSA (*n*=6). 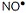 released by S-NO-HSA was quantified using a porphyrinic nanosensor (for details see Supplementary Fig. 16 and methods).

We next applied different 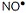 donors to visualize and compare 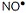 dynamics on the single cell level (Fig. 3). For this purpose, we used low-molecular-weight 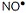 donors and *S*-nitroso human serum albumin (S-NO-HSA) with a high capacity to stably release 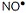 over time, due to its long half-life^31^. While 30 s perfusion of HeLa cells with NOC-7 and SNP evoked almost identical cellular 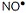 signals, PROLI NONOate, a more instable compound^32^, led to a more transient 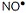 increase, with the highest peak under these conditions (Fig. 3a). In HeLa cells, addition of NOC-7 to the image medium induced clear variances of the strength of 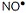 signals within cells on the same dish, while the average responses among different dishes were nearly homogeneous (Fig. 3b). These findings might point to cell-to-cell heterogeneities in the 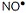 scavenging capacity of HeLa cells. Addition of S-NO-HSA induced a distinctly slower increase of cellular 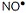 levels compared with the fast 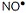-liberating low-molecular-weight 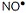 donors (Fig. 3c; Supplementary Fig. 15, left panel). SNP, which is known to liberate 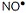 by reacting with biomolecules in the cell^23^, increased cellular 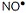 levels only at high concentrations (≥1 mM; Supplementary Fig. 15, right panel), pointing to a weak capacity of this compound to release 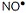. These experiments demonstrate that geNOps enable the precise characterization of highly diverse 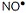 donors by providing a reliable, real-time readout of the actual 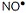 dynamics on the single-cell level in response to these compounds. Such information is valuable for an efficient testing of newly developed, 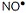-releasing and 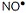-scavenging drugs. On the basis of the capacity of S-NO-HSA to stably release constant amounts of 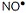, this compound was further used to estimate the concentration reflected by geNOps signals. For this purpose, the free 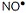 concentrations released by different concentrations of S-NO-HSA were determined using a highly sensitive 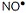 porphyrinic nanosensor (Supplementary Fig. 16) and plotted against respective geNOp responses (Fig. 3d). This analysis was further used to estimate the physiological 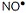 concentration in single endothelial cells. Moreover, the approach was used to estimate the on and off kinetics of C-geNOp to respond to 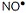 (Supplementary Note 2).

### Correlations of 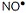 signals with cell functions

**Figure 4:**
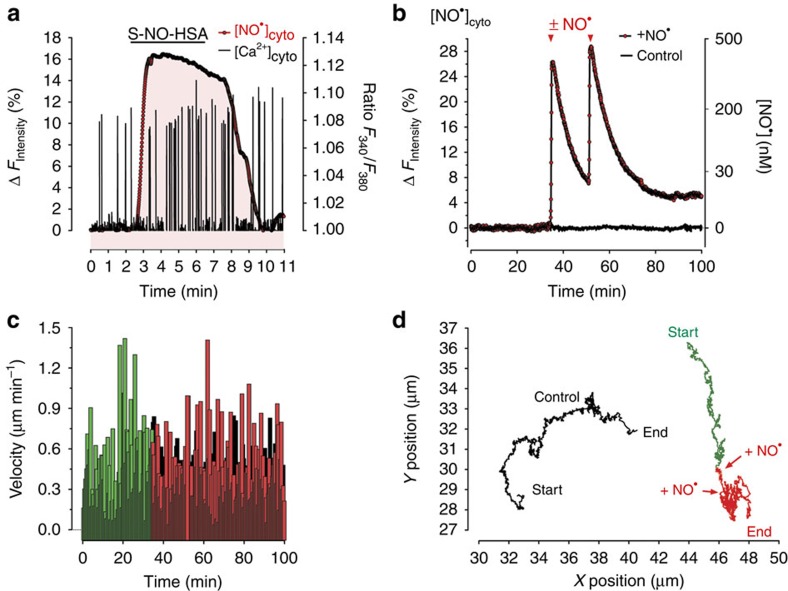
Live-cell imaging of 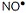 signals and cell functions in primary cardiomyocytes and glioblastoma cells using geNOps. (**a**) Curves represent representative simultaneous recordings of cellular Ca^2+^ (black ratio curve) and 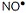 (red inverted curve) signals over time of a single fura-2/am-loaded embryonic ventricular cardiomyocyte expressing G-geNOp. The cell was treated with 30 μM S-NO-HSA in the presence of extracellular Ca^2+^ using a perfusion system (*n*=4). (**b**) Representative recordings of cellular 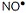 dynamics (red inverted curve, *n*=4) of human glioblastoma cells (U87-MG cells) expressing C-geNOp. Cells were either treated with a mixture of 10 μM PROLI NONOate and 10 μM NOC-7 (red curve) or remained untreated (control cell, black curve). (**c**) Cell velocity of glioblastoma cells in μm min^−1^ extracted from the *X*/*Y* positions over time of a control cell (black columns) and a cell treated with 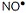 donors as indicated in **b** and **d**. (**d**) Graphs represent *X*/*Y* positions of glioblastoma cells over time as indicated in **b** and **c**.

To further demonstrate the applicability of geNOps in other cell types, the probes were expressed in primary embryonic ventricular cardiomyocytes. By measuring geNOps signals, we could show that the addition of nitric oxide donors allowed us to evoke controllable cellular 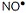 elevations in this cell type (Supplementary Fig. 17). Hence, we further used this approach to mimic and investigate the paracrine effect of exogenously generated 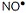 on spontaneous Ca^2+^ signals in single cardiomyocytes. Elevation of 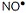 did not prevent Ca^2+^ transients but temporally correlated with a moderate increase of the frequency of Ca^2+^ oscillations (Fig. 4a), confirming that 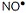 is a regulator of myocardiac function^33^. In an additional set of experiments, we used the geNOps technology to relate elevated cellular 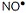 levels with the motility of individual glioblastoma cells (Fig. 4b–d). Short treatment of the cells with a mixture of PROLI NONOate and NOC-7 highly increased the cellular 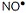 concentration (Fig. 4b). This procedure did not affect the overall cell motility (Fig. 4c) but markedly reduced the radius of cell movements (Fig. 4d), indicating that high 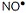 pulses might impair the metastatic spread of glioblastoma cells.

### Imaging of Ca^2+^-induced 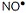 formation in endothelial cells

**Figure 5:**
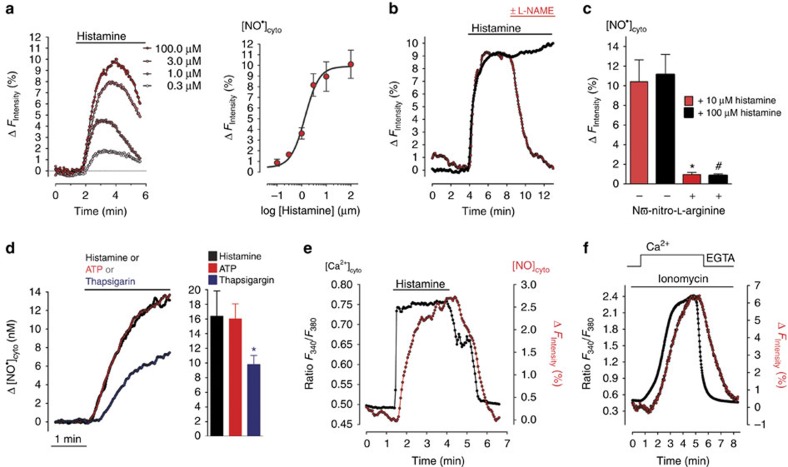
Live-cell imaging of Ca^2+^-triggered 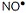 production in signals endothelial cells. (**a**) Single endothelial cell (EA.hy926 cells) 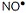 responses upon cell treatment with different concentrations of histamine (right panel, 0.3 μM; 1.0 μM; 3.0 μM; 100 μM histamine, inverted curves are shown) in the absence of extracellular Ca^2+^. For the concentration response curve (right panel), cells expressing C-geNOp were stimulated with 0.1 μM (*n*=6), 0.3 μM (*n*=6), 1.0 μM (*n*=7), 3.0 μM (*n*=7), 10.0 μM (*n*=7) or 100.0 μM (*n*=12) histamine, yielding an effector concentration for half-maximum response of 1.4 (0.8–2.5) μM. Red points represent average values±s.e.m. (**b**) Cellular 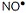 dynamics of EA.hy926 cells expressing C-geNOp. Cells were stimulated with 100 μM histamine in Ca^2+^ containing buffer for 9 min under control conditions (black inverted curve, *n*=4) or during stimulation, 1 mM L-NAME was added (red inverted curve, *n*=9). (**c**) Columns represent maximal G-geNOps signals±s.e.m. in response to either 10 (red columns) or 100 μM (black columns) histamine under control conditions (*n*=5 for both histamine concentrations) and in the presence of the NOS inhibitor (1 mM; *n*=10 for both histamine concentrations). *P<0.05 versus control (10 μM histamine); #*P*<0.05 versus control (100 μM histamine). *P* values were calculated using unpaired *t*-test. (**d**) Average 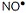 curves over time (right panel) and statistics of the maximal cytosolic 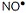 increase (columns representing average values±s.e.m. in the left panel) in EA.hy926 cells in response to 30 μM histamine (black curve, black column, *n*=16), 30 μM ATP (red curve and red column, *n*=20) or 1 μM thapsigargin (blue curve, blue column, *n*=15). Endothelial cells expressing C-geNOps were used *P<0.05 versus histamine/ATP using unpaired *t*-test. (**e**) Curves represent simultaneous recordings of cellular Ca^2+^ (black ratio curve) and 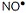 (red inverted curve) signals over time of a single fura-2/am-loaded endothelial cell expressing O-geNOp as shown in Supplementary Fig. 21. The cell was stimulated with 100 μM histamine in the presence of extracellular Ca^2+^. (**f**) Simultaneous recordings of cellular Ca^2+^ (black ratio curve) and 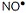 (red inverted curve) signals over time of a single fura-2/am-loaded endothelial cell expressing G-geNOp. During imaging, the cell was treated with 1 μM ionomycin in the absence (1 mM EGTA) and presence of 2 mM Ca^2+^.

We tested the utility of geNOps in visualizing physiologically triggered, Ca^2+^-activated enzymatic 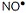 generation in the human umbilical vein cell line EA.hy926, which is known to solidly express the endothelial nitric oxide synthase (eNOS)^34^. Ca^2+^ mobilization with different concentrations of the physiological inositol 1,4,5-trisphosphate (IP_3_)-generating agonist histamine resulted in clear responses of functional (Fig. 5a), but not mutated geNOps (Supplementary Fig. 18), demonstrating endogenous Ca^2+^-triggered concentration-dependent 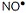 production in single endothelial cells. The 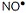 signals in endothelial cells were reduced in the absence of Ca^2+^ entry (Supplementary Fig. 19), confirming the importance of Ca^2+^ influx for sustained eNOS activity^35^. Moreover, as expected the histamine-evoked 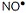 signals were strongly diminished in the presence of NOS inhibitors (Fig. 5b,c; Supplementary Fig. 20). While cell treatment either with the IP_3_-generating agonist histamine or ATP induced almost identical patterns of 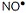 elevations, the sarco/endoplasmic reticulum Ca^2+^-ATPase (SERCA) inhibitor thapsigargin evoked a clearly delayed, slower and weaker 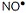 rise in endothelial cells (Fig. 5d). To correlate the temporal patterns of cytosolic 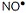 and Ca^2+^ dynamics in individual cells, red-shifted geNOps (either G-geNOp or O-geNOp) were co-imaged with fura-2, an ultraviolet excitable chemical Ca^2+^ indicator^36^ (Fig. 5e,f). This approach unveiled a temporal delay and slower kinetics of cellular 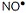 dynamics compared with respective cytosolic Ca^2+^ signals elicited by addition of either histamine (Fig. 5e; Supplementary Fig. 21) or the Ca^2+^ ionophore ionomycin (Fig. 5f). However, these experiments also highlighted a strict correlation between the enzymatic 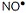 production and cytosolic Ca^2+^ signals in single endothelial cells.

### Imaging of 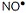 within mitochondria of endothelial cells

**Figure 6:**
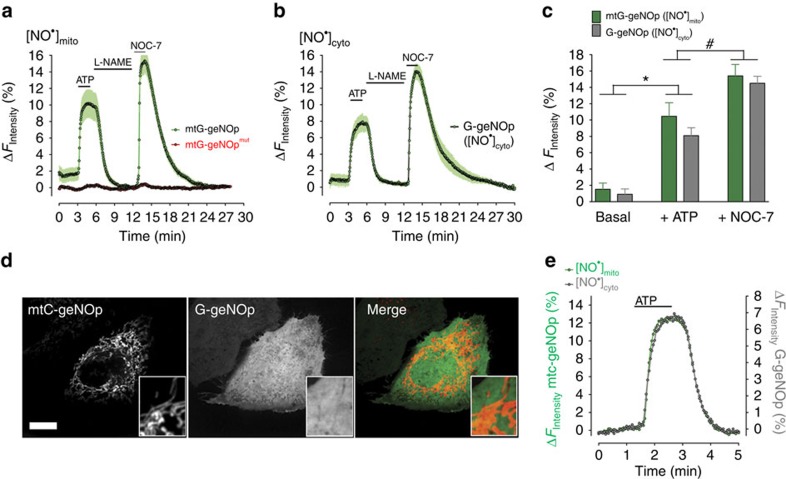
Visualization of 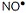 signals within mitochondria of signals endothelial cells. (**a**) Average curves±s.e.m. showing mitochondrial 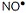 signals measured with mtG-geNOp expressed in EA.hy926 cells (green curve, *n*=7) and respective signals obtained with mtG-geNOp^mut^ (red curve, *n*=7). Cells were treated first with 100 μM ATP, then with 1 mM L-NAME and subsequently with 10 μM NOC-7. (**b**) Average curves±s.e.m. showing cytsolic 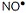 signals measured with G-geNOp expressed in EA.hy926 cells (green curve, *n*=5). As shown in **a**, cells were treated first with 100 μM ATP, then with 1 mM L-NAME and subsequently with 10 μM NOC-7. (**c**) Columns represent maximal average values of curves shown in **a** and **b**. *P<0.05 versus basal. **#***P*<0.05 versus +ATP. *P* values were calculated using unpaired *t*-test. (**d**) Confocal images of endothelial cells expressing both mtC-geNOp (left image) and cytosolic G-geNOp (middle image). Scale bar, 10 μm. (**e**) Representative simultaneous recordings of mtC-geNOp (grey curve) and cytosolic G-geNOp (green curve) signals over time in a single EA.hy926 cell in response to 100 μM ATP.

Next, we used endothelial cells expressing mitochondria-targeted G-geNOp to test whether endogenously generated 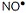 is detectable within these organelles. Cell treatment with ATP elicited clear mtG-geNOp signals, which were strongly reduced by the addition of L-NAME and recovered robustly in the presence of NOC-7 (Fig. 6a,c). The fluorescence of the 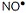-insensitive mtG-geNOp^mut^ did, however, not respond to any of these treatments under the same experimental conditions (Fig. 6a). Respective geNOps signals in endothelial cells expressing non-targeted cytosolic G-geNOp did not significantly differ from the mitochondrial responses (Fig. 6b,c) These data demonstrated that NOS activation upon Ca^2+^ mobilization with an IP_3_-generating agonist also yield a significant elevation of 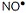 within mitochondria in single endothelial cells. Next, we performed multichannel imaging of mitochondria-targeted and cytosolic geNOps in the same single cells to correlate 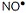 signals within both compartments. While the fluorescence of mtC-geNOp could be completely separated from the fluorescence of cytosolic G-geNOp using confocal microscopy (Fig. 6d), a spectral overlay between ECFP- and EGFP-based geNOps was observed using a wide-field imaging system (Supplementary Fig. 22). Hence, we applied spectral unmixing^37^, which eliminated the spectral crosstalk between mitochondria-targeted and cytosolic geNOps (Supplementary Fig. 22; Supplementary Note 3). To validate this procedure, endothelial cell co-expressing C-geNOp^mut^ and mtG-geNOp were treated first with ATP and subsequently with NOC-7. Neither ATP nor NOC-7 significantly affected the fluorescence of the non-targeted cytosolic C-geNOp^mut^, while in the same cell the mitochondria-targeted mtG-geNOp showed clear responses, confirming complete separation of respective fluorescence channels (Supplementary Fig. 23). Co-imaging of mtC-geNOp and cytosolic G-geNOp revealed identical ATP-triggered 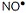 signals in both compartments of a single individual endothelial cell (Fig. 6e). The same result was obtained in cells expressing both mtG-geNOp and cytosolic C-geNOp (Supplementary Fig. 23). These data indicate that upon eNOS activation 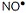 instantly and efficiently increases both in the cytosol and within the mitochondrial matrix. In addition, our data demonstrate that upon removal of the agonist, 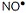 declines with the same kinetics in both compartments (Fig. 6e; Supplementary Fig. 23).

## Discussion

Although the importance of 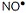 as a key regulator of diverse cell functions is well accepted, little is known about the actual dynamics of this radical within single cells and subcellular compartments^8^. The lack of practicable techniques that provide a selective, direct and real-time readout of single (sub)cellular 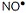 dynamics hampered investigations in this regard^38^, since 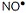 has been discovered to function as an endothelium-derived relaxing factor in 1987 (ref. 39). The differently coloured geNOps, we have introduced in this study, can be used for real-time tracking of 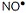 in single cells and subcellular compartments such as mitochondria. The key feature of geNOps is that these probes selectively bind 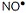, which induces a significant quenching of the intensity of the FP within the probe. This concentration-dependent effect occurs immediately upon 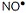 binding and is fully reversible and repeatable so that geNOps can be used to visualize (sub)cellular 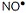 signals dynamically and over a long period of time.

Convincing measurements of single cell 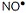 signals in real time with other small chemical fluorescent 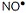 indicators such as 4,5-diaminofluorescein diacetate have not been accomplished so far. While such probes can be easily loaded into cells, 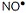 and other reactive species irreversibly modify the chemical structure of these fluorescent indicators so that they do not provide a selective and actual readout of cellular 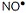 signals^40^. Moreover, small chemical 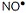 probes have been shown to be cytotoxic and can aggregate within certain cell compartments, both of which considerably limit their range of usability^40^,^41^. Hence, it is very important to develop novel improved 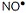 probes that overcome these limitations. In contrast to small chemical indicators, genetically encoded fluorescent probes are usually not toxic for cells and can be efficiently localized to virtually any subcellular compartments^42^,^43^. The development of protein-based sensors is, however, challenging^44^. Usually, this requires fusion of proper sensing domains to one or more FPs in a way that a measurable signal can be obtained upon the specific binding of the analyte of interest. While we used a well-characterized bacteria-derived 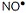-binding domain to generate functional fluorescent geNOps, Pearce *et al*. used metallothionein, a cysteine-rich small protein with unknown functions, to detect the production of 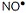 in intact cells^45^. In their study, the authors could confirm that 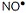 interacts with metallothionein, and that 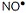 binding affects the protein conformation, which results in increased Förster resonance energy transfer (FRET) between terminally located FPs. However, this FRET-based probe only provides a readout of a single 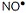 elevation, as it does not respond to 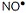 in a reversible manner. Moreover, the probe releases metal ions upon 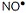 binding that might impact cell functions. Actually, only few 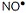-binding proteins in mammalians, plants and bacteria have been identified and characterized so far. Accordingly, the number of known putative 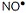-sensing domains for the development of protein-based 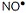 probes is quite limited. In mammalians, the soluble guanylate cyclase (sGC) is the dominant 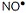 responsive target, which reversibly binds 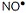 via a haem iron centre^46^. 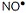 binding to sGC stimulates the generation of cyclic GMP (cGMP), an intracellular second messenger that regulates multiple cell signalling pathways^47^. Sato *et al*. generated an indirect 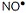 probe based on 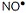 binding to sGC and the subsequent cGMP determination via a FRET-based sensor^15^. Although this probe was used to image 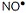 in the low nano molar range, the technique has some limitations. First of all, the fluorescent probe has a small dynamic range, measures cGMP and not 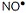 directly. In addition, the practicality of the usage of this sensor is rather poor as it depends on the simultaneous expression of two different constructs, which have to dimerize to form the working probe. As the dimerization of the alpha and beta subunit of the sGC is essential for 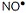 binding to the haem iron centre of this protein, we considered sGC as a suboptimal candidate for the development of fluorescent geNOps that directly sense 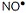.

In line with a recent study that showed the importance of iron(II) in the non-haem 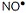-binding domain of norR for the functionality of this bacterial transcription factor^17^, our data clearly indicate that sufficient iron(II) within the bacteria-derived GAF domain of geNOps is essential to obtain full 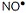 responsiveness of all the differently coloured probes. Iron(II) supplementation was essential to significantly increase the dynamic range of all geNOps in different cell types. We established a fast, simple and non-harmful treatment to supply geNOps-expressing cells with efficient amounts of iron(II), which under normal cell culture conditions is provided rather poorly^48^. While iron(II) supplementation did not cause any obvious problems when using the geNOps technology in cultured cells, this procedure might limit the applicability of geNOps. It might be challenging to increase the iron(II) amount of expressed geNOps when using this technology *in vivo*. On the other hand, the iron(II) homeostasis in living organisms might be anyway sufficient to supply expressed geNOps with iron(II) adequately. However, further experiments are necessary to investigate whether or not geNOps are useful tools to image 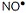 signals also *in vivo*.

The basal fluorescence of geNOps was affected by pH changes as FPs are pH sensitive^19^. However, the responsiveness of geNOps to 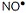 remained over a huge pH range, indicating that these probes can be also used in alkaline and acidic compartments such as mitochondria or endo- and lysosomes, respectively. Indeed, we could demonstrate that mitochondria-targeted geNOps remain fully functional. Nevertheless, due to the pH sensitivity of FPs, acute pH changes within cells^49^ might complicate correct interpretation of geNOps signals. In this study, we, hence, performed key experiments using mutated probes that did not respond to 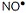, but kept their pH sensitivity. Using these probes under the same experimental conditions allowed us to estimate that the geNOps signals reflect real (sub)cellular 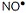 dynamics and were not due to acute pH changes. The development of novel optimized geNOps that contain other bright and pH-stable FPs would be a direct approach to circumvent this problem. Considering the high number of additionally available and newly developed FP variants^19^ with improved properties as well as novel techniques to generate and test whole libraries of altered probes^20^, such efforts will certainly yield in advanced geNOps in near future.

Due to the high signal-to-noise ratio of geNOps, we were able to study both the dynamics of (sub)cellular 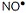 signals in response to even low concentrations of different 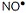 donors and endogenously Ca^2+^-triggered 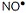 production in endothelial cells. Our experiments revealed that Ca^2+^ mobilization using the two different IP_3_-generating agonists histamine and ATP evoked identical 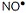 increases in endothelial cells, while the SERCA inhibitor thapsigargin was less effective to elevate 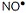 production. These results are consistent with other reports that show clear differences in the kinetics and amplitude of cytosolic Ca^2+^ signals in response to either IP_3_-generating agonists or SERCA inhibitors^50^,^51^,^52^. The combination of fura-2 with red-shifted geNOps demonstrated that Ca^2+^ signals temporally correlate with respective 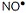 transients in endothelial cells. These findings point to a fast on and off kinetic of the Ca^2+^-regulated eNOS activity and displayed how tight this enzyme is under the control of the cytosolic Ca^2+^ concentration. Targeting geNOps into the mitochondrial matrix in combination with cytosolic geNOps enabled us to simultaneously monitor 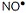 dynamics in both compartments in single individual endothelial cells. These experiments showed that Ca^2+^-triggered 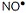 signals are identical in both compartments, confirming the high capability of 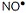 to diffuse across biomembranes. It has been suggested that mitochondria are able to generate 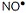 autonomously under certain conditions^53^. Moreover, the existence of NOS located within mitochondria has been proposed, while the respective protein has not been identified explicitly so far^54^. Our experiments shown in this manuscript neither confirm nor argue against a mitochondrial NO production, but the geNOps technology will be very useful to further investigate this and other remaining important question in the field of 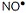-related cell biology.

In summary, we have generated differently fluorescent geNOps and have demonstrated their suitability to single-live-cell 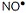 imaging in different cell types. These novel tools will enhance the high-resolution investigation of intracellular 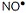 generation, degradation, as well as diffusion under physiological and pathological conditions. This, in turn, will improve our understanding of the complex cellular metabolism and signalling patterns of one of nature's most reactive and versatile messengers.

## Methods

### Cloning of geNOps

Briefly, cloning was performed according to standard procedures and all products were verified by sequencing. Genomic DNA of *E. Coli* DH10α was isolated by a DNA extraction protocol using phenol/chloroform extraction followed by ethanol precipitation and subsequent solubilization in 30 μl deionized water. The bacterial DNA was used as a template to isolate the GAF subunit of the NorR transcription factor in a PCR with the following primers: forward 5′-GGCATCGATATGAGTTTTTCCGTTGATGTGC-3′ that adds a ClaI restriction site and reverse 5′-GGCAAGCTTAAGGGGACAAGCCAATCATCT-3′ including a stop codon and a HindIII site. To obtain various single FP-based geNOps, the PCR product of the GAF domain was C terminally fused to a super ECFP, a blue-green emitting FP (GEM)^20^, an EGFP, a circularly permuted Venus or a mKO_k_ via ClaI and HindIII in a mammalian expression vector pcDNA3.1(-) (Invitrogen, Austria). To construct the 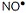-insensitive probes (C-geNOp^mut^ and G-geNOp^mut^), the two argingines at positions 75 and 81 of the GAF domain were mutated by a two-step PCR protocol using two additional primers forward 5′-AGCGCTGGAAGCGATTGCCGCCG-3′ and reverse 5′-CCGGCGGCGGCAATCGCTTCCAGCGCT-3′. For targeting geNOps into mitochondria, two COX VIII mitochondria-targeting sequences were added to the N terminus of respective constructs. To target C-geNOp to the outer surface of the plasma membrane, a membrane leading sequence of the human cadherin 13 (24 amino acids) was added to the N terminus and the GPI-anchor sequence of cadherin 13 (coding for 26 amino acids) were fused to the C terminus of C-geNOp, respectively.

### Chemicals and buffer solutions

Cell culture materials were obtained from PAA laboratories (Pasching, Austria). Histamine hydrochloride, Iron(II)fumarate, 2,5-Di-t-butyl-1,4-benzohydroquinone, ethylene glycol tetraacetic acid (EGTA), Tris-HCl, monensin, nigericin, CORM-3, L-NAME and potassium superoxide were purchased from Sigma Aldrich (Vienna, Austria). NOC-7 and PROLI NONOate were from Santa Cruz (San Diego, USA). ATP was obtained from Roth (Graz, Austria). Peroxynitrite was from Cayman Chemical (Michigan, USA). SNP was purchased from Gatt-Koller (Absam, Austria). Ionomycin was obtained from Abcam (Cambridge, UK).

Before the experiments, cells were washed and maintained for 20 min in a HEPES-buffered solution (storage buffer) containing 138 mM NaCl, 5 mM KCl, 2 mM CaCl_2_, 1 mM MgCl_2_, 1 mM HEPES, 2.6 mM NaHCO_3_, 0.44 mM KH_2_PO_4_, 0.34 mM Na_2_HPO_4_, 10 mM D-glucose, 0.1% vitamins, 0.2% essential amino acids and 1% penicillin–streptomycin, the pH was adjusted to 7.4 with NaOH.

During the experiments, cells were perfused in a physiological Ca^2+^-containing buffer (Ca^2+^ buffer), which consisted of 140 mM NaCl, 5 mM KCl, 2 mM CaCl_2_, 1 mM MgCl_2_, 10 mM D-glucose and 1 mM HEPES, the pH was adjusted to 7.4 with NaOH. For Ca^2+^-free experiments 1 mM EGTA was added to the perfusion buffer instead of 2 mM Ca^2+^. Preparation of iron(II) fumarate solution was performed in the Ca^2+^ buffer by adding 1 mM iron(II) fumarate and 1 mM ascorbic acid and stirring at room temperature in the dark. During the experiments, various NO donors or other pharmacological compounds were applied to the cells using a gravity-based perfusion system connected with a conventional vacuum pump (Chemistry diaphragm pump ME 1C, Vacuubrand, Wertheim, Germany).

### Measurement of 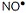 release using a poryphyrinic nanosensor

For estimation of 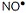 concentrations, release of 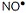 from S-NO-HSA dissolved in physiological saline was measured with a poryphyrinic nanosensor in a tissue culture bath at identical concentrations as used for geNOp signal imaging. The nanosensor was operated in a three-electrode system, consisting of the sensor working electrode, a platinum wire (0.1 mm) counter electrode, and a standard calomel reference electrode. The current proportional to concentration was measured by the nanosensor operated in an amperometric mode at a constant potential of 0.65 V. The response time of the nanosensors was 0.1 ms. The 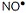 nanosensor was calibrated for the range 1 μmol·L^−1^ using aliquots of a 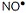 standard-saturated aqueous solution (1.76 mmol l^−1^). The amperometric signals for NO were recorded with a computer-based Gamry VF600 voltametric analyser.

Equation for [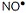]_cyto_ from respective changes in fluorescence intensities of C-geNOps (Δ*F*) was obtained by plotting the respective 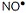 concentrations (obtained with the poryphyrinic nanosensor) against Δ*F*_Intensity_ values and fitting the data with a saturation kinetic:


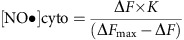


where *K* is the concentration of S-NO-HSA at half maximal response (4.50) and Δ*F*_max_ is the maximal geNOp response (19.16).

### Cell culture, transfection and fura-2/AM loading

HeLa cells were grown in DMEM (Sigma Aldrich) containing 10% fetal bovine serum, 100 U ml^−1^ penicillin and 100 μg ml^−1^ streptomycin. Culture medium of EA.hy926 cells contained additionally 1% HAT (5 mM hypoxanthin, 20 μM aminopterin and 0.8 mM thymidine). Human glioblastoma U87-MG cells were cultured in DMEM supplemented with 10% fetal bovine serum, 4 mM glutamine, 50 U ml^−1^ penicillin and 50 mg ml^−1^ streptomycin. At 60–80% confluence, cells in 30-mm imaging dishes were transfected with 1 ml of serum- and antibiotic-free medium that had been mixed with 1.5 μg of the approprioate plasmid DNA and 3 μg of TransFast transfection reagent (Promega). Cells were maintained in a humidified incubator (37 °C, 5% CO_2_, 95% air) for 16–20 h before changing back to the respective culture medium. All experiments were performed either 24 or 48 h after transfection. For dual recordings using fura-2, cells were incubated in storage buffer containing 3.3 μM fura-2/AM for 40 min. Before the experiments, cells were incubated 10 min in the iron(II) fumarate solution.

### Culturing embryonic chicken ventricular cardiomyocytes

Ventricular myocytes were isolated from embryonic chick hearts. The hearts of 7-day embryos were removed, and the ventricles were chopped off, minced and transferred to a nominally Ca^2+^- and Mg^2+^-free Hanks' balanced salt solution (HBSS; in mM: 137 NaCl, 5.4 KCl, 0.34 Na_2_HPO_4_, 0.44 KH_2_PO_4_, 4.2 NaHCO_3_ and 5 glucose, pH 7.4) containing 0.25% trypsin (bovine pancreas, Sigma-Aldrich). The suspension was transferred to a shaker bath at 37 °C for 7 min, afterwards cells were released with mechanical disruption (pipetting) and filtered through a 100-μm mesh. HBSS, supplemented with fetal calf serum (5% final concentration), was added to stop trypsin activity. The cell suspension was centrifuged at 100*g* for 5 min at 4 °C, the supernatant was discarded and the cell pellet was resuspended in fresh trypsin-free HBSS. The centrifugation and resuspension processes were then repeated. After the third time cells were resuspended in M199 cell culture medium (Sigma-Aldrich, supplemented with 4% fetal calf serum, 2% horse serum and 0.7 mM glutamine, pH 7.4) to yield a density of 3.5 × 10^5^ cells per ml.

### Live-cell imaging of 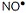 concentrations with geNOps

Measurements were performed on two different wide-field imaging systems: an inverted and advanced fluorescent microscope with a motorized sample stage (Till Photonics, Graefling, Germany) was used. The probes were excited via a polychrome V (Till Photonics), and emission was visualized using a × 40 objective (alpha Plan Fluar 40 ×, Zeiss, Göttingen, Germany), and a charge-coupled device camera (AVT Stringray F145B, Allied Vision Technologies, Stadtroda, Germany). C-geNOp and M-geNOp were excited at 430 nm, G-geNOp and Y-geNOp at 480 nm, and O-geNOp at 515 nm. Emitted light was collected with emission filters CFP emitter 482/18 nm, yellow fluorescent protein emitter 514/3 nm or orange fluorescent protein-emitting filter (560dcxr), respectively. In addition, for simultaneous measurements of cytosolic Ca^2+^, Fura-2 was alternately excited at 340 and 380 nm, and emissions were captured at 515 nm (515dcxr). For control and acquisition, the Live acquisition 2.0.0.12 software (Till Photonics) was used.

Alternatively, geNOps were visualized on a Nikon eclipse TE300 inverted microscope (Tokyo, Japan) using a × 40 objective (Plan Fluor, Nikon, Vienna or Fluor, Zeiss, Jena, Germany) and fluorescence was recorded with a Spot pursuit charge-coupled device camera (Visitron Systems, Puchheim, Germany). Fura-2 and geNOps were excited as described above, and emissions were collected using emission filter 510WB40 or XF56 (Omega Opticals, Brattleboro, VT, USA). Data acquisition and control were done using the VisiView Premier Acquisition software (Visitron Systems).

### Characterization of the pH sensitivity of geNOps

To characterize the pH sensitivity, HeLa cells expressing C-geNOp were treated using a series of buffers with various pH values ranging from 5 to 9. Cells were prepared with 10 μM nigericin and 10 μM monensin, and 20 mM MES (for pH 5–6.5), 20 mM HEPES (for pH 7–7.5) or 20 mM Tris-HCl (for pH 8–9) containing buffer. Cells were additionally stimulated with 10 μM NOC-7 at respective pH values.

### Construction of structural models of geNOps

Models of all geNOps were constructed with the online tool Phyre2 (Protein Homology/analogy Recognition Engine V 2.0). Analyses of the predicted proteins were performed with the software DeepView/Swiss Pdb viewer V4.1.0 observed from ExPASy.

### Cell velocity measurements

Centre of mass was determined for cells over the whole stack after binearization with an Otzu auto threshold in ImageJ. To determine the cell velocity between consecutive positions, following equation was used:


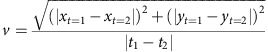


(*x*) and (*y*) are the localization coordinates of the centre of mass at consecutive time points (*t*_1_) and (*t*_2_).

### Statistical analysis

Statistical analysis was performed using the GraphPad Prism software version 5.04 (GraphPad Software, San Diego, CA, USA). Analysis of variance and *t*-test were used for evaluation of the statistical significance. *P*<0.05 was defined to be significant. At least three different experiments on different days have been performed for each experimental set-up.

## Additional information

**How to cite this article:** Eroglu, E. *et al*. Development of novel FP-based probes for live-cell imaging of nitric oxide dynamics. *Nat. Commun.* 7:10623 doi: 10.1038/ncomms10623 (2016).

## Supplementary Material

Supplementary Information Supplementary Figures 1-23, Supplementary Table 1 and Supplementary Notes 1-3

Supplementary Movie 1 NOC-7-induced fluorescence quenching of O-geNOp expressed in a HeLa cell. The video represents an original measurement of dynamic changes of O-geNOp fluorescence over time upon cell treatment with 10 µM NOC-7. The NO• donor was added and removed via a gravity-based perfusion system as shown in the left panel. Changes in fluorescence intensity of the HeLa cell expressing O-geNOp is shown in pseudo color, grey scale, and as a XY-plot of fluorescence intensity over time
