# Supplementary material for: Development of novel FP-based probes for live-cell imaging of nitric oxide dynamics
Source: Nat Commun. 2016 Feb 4;7:10623. doi: 10.1038/ncomms10623 (PMC4743004; doi:10.1038/ncomms10623)
Supplement: Supplementary Information — Supplementary Figures 1-23, Supplementary Table 1 and Supplementary Notes 1-3 [file ncomms10623-s1.pdf]

Supplementary Figure 1 I The expression efficiency of geNOps in HeLa cells does not differ from that of other constructs.

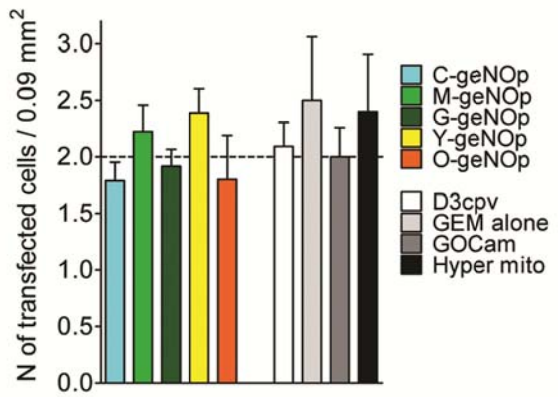

Columns represent average numbers of transfected HeLa cells per 0.09 mm^2. Imaging was performed using a 40x objective at binning 4 using a fully automated imaging system. C-geNOp (n=19) and M-geNOp (n=18) were excited at 430 nm, G-geNOp (n=12) and Y-geNOp (n=18) were excited at 480 nm, and O-geNOp (n=10) at 575 nm. The cameleon D3cpv (n=11) containing eCFP and YFP and the circularly permuted green emitting FP (GEM, n=6) were illuminated at 430 nm. The red-shifted cameleon GOCam (n=6) containing cpGFP and OFP and the H_2O_2 probe Hyper (n=5) were excited at 480 and 430 nm, respectively.

Supplementary Figure 2 I Pretreatment of different cell types with iron(II) fumarate and vitamin C neither affects cell morphology nor viability nor metabolic activity

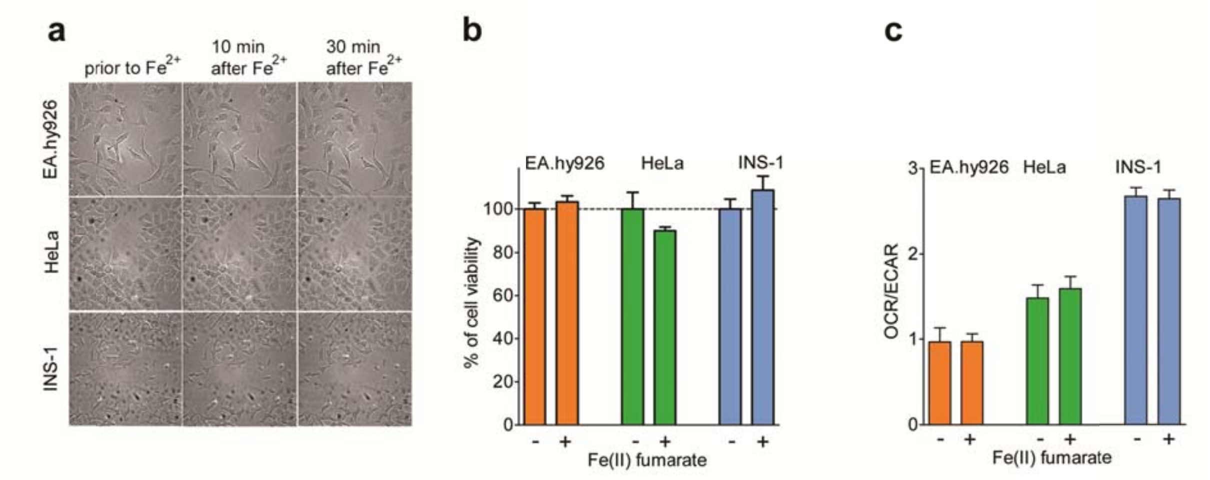

(**a**) Representative wide field images (n=10 for all conditions) of endothelial cells (upper panel), HeLa cells (middle panel) and INS-1 cells (lower panel) prior to the treatment with 1 mM iron(II) fumarate and vitamin C (left images), 10 minutes after the addition of iron(II) / vitamin C (middle images) and 30 minutes thereafter (right images). Images were taken using a 10x objective (Nikon). (**b**) Columns represent cell viability of INS-1 cells (n=8), HeLa cells (n=7) and EA.hy926 cells (n=8). Without treatment (white columns) and after treatment with 1mM iron(II)-fumarate / vitamin C (black columns). Average values were calculated as percentage of cell viability of the corresponding untreated control cells. (**c**) OCR/ECAR ratios of INS-1 cells, HeLa cells, and EA.hy926 cells without treatment (white columns; INS-1 cells n = 10; Hela cells n=12; EA.hy926 cells n=7) and after 10 minutes incubation with 1mM iron(II)-fumarate / vitamin C (black columns; INS-1 cells n = 21; Hela cells n=16; EA.hy926 cells n=11).

Supplementary Figure 3 I All geNOps show linear correlations between basal fluorescence and NO•-induced fluorescence quenching

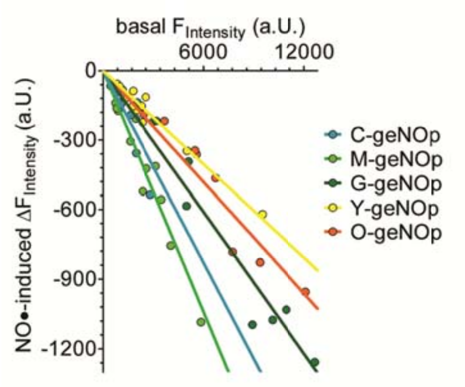

Circles represent single values of NO•-induced fluorescence quenching (Y-axis) in respect to basal fluorescence intensities (X-axis) of the differently colored geNOps. HeLa cells expressing geNOps were stored in a buffer containing 1 mM iron(II) fumarate and 1 mM vitamin C for 10 minutes prior to microscopy. During imaging 10 µM NOC-7 was applied for 2 minutes as shown in Fig.1 c. Lines represent respective linear regression fits. C-geNOp (R^2=0.7553), M-geNOp (R^2=0.9359), G-geNOp (R^2=0.9270), Y-geNOp (R^2=0.9599), and O-geNOp (R^2=0.9264).

Supplementary Figure 4 I G-geNOp shows long-term reproducible stable signals in response to consecutive applications of the NO• donor SNP

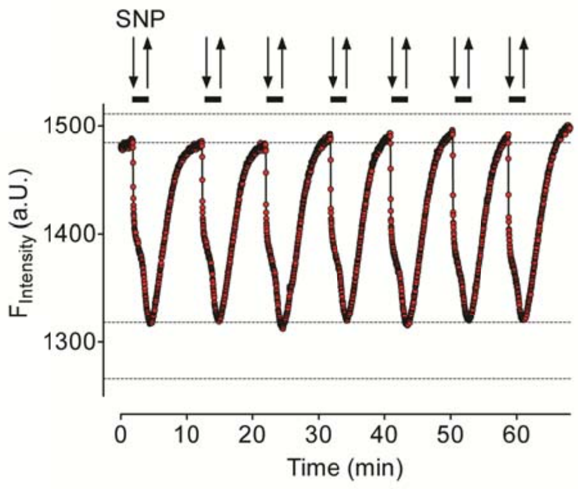

Representative curve showing G-geNOp fluorescence over time upon the consecutive application and removal of 5 mM SNP. HeLa cells expressing G-geNOp were excited at 480 nM and emission was collected at 510 nm, respectively.

Supplementary Figure 5: I Oxidation of iron(II) to iron (III) within G-geNOp by H_2O_2 reduces the NO•-induced quenching effect

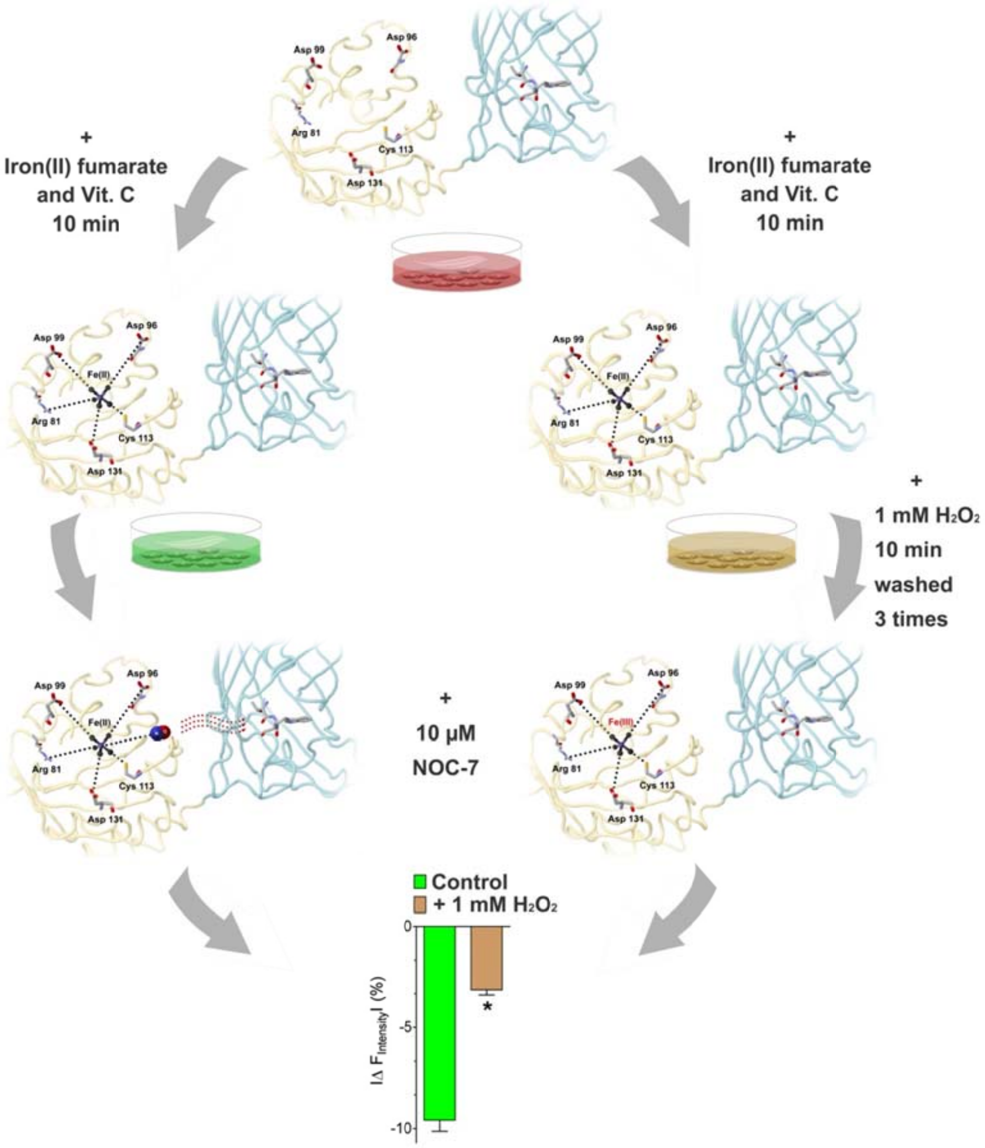

Schematic illustration of the experimental design to test the impact of oxidizing hydrogen peroxide (H_2O_2) on geNOps in HeLa cells. Cells expressing G-geNOp (red petri dish) were first incubated with a buffer containing 1 mM iron(II) fumarate and 1 mM ascorbic acid for 10 minutes to supply the non-heme iron(II) center of the NO• probe with sufficient iron(II). Cells were then washed and either kept in a standard experimental buffer (green petri dish) or incubated with 1 mM H_2O_2 for further 10 minutes (brown petri dish). During this procedure H_2O_2 oxidizes iron (II) (Fe^{2+}) to iron (III) (Fe^{3+}). Prior to fluorescence microscopy cells were washed three times with the experimental buffer. During imaging 10 µM NOC-7 was added for 5 minutes using a perfusion system. Bars illustrate the NOC-7 induced quenching of G-geNOp under control conditions (green column, n= 11) or upon pre-treatment with 1 mM H_2O_2 (brown column, n=10). mean ± SEM. *$P < 0.05$ vs. Control.

Supplementary Figure 6 I Boosting the responsiveness of geNOps to NO• by iron(II) supplementation

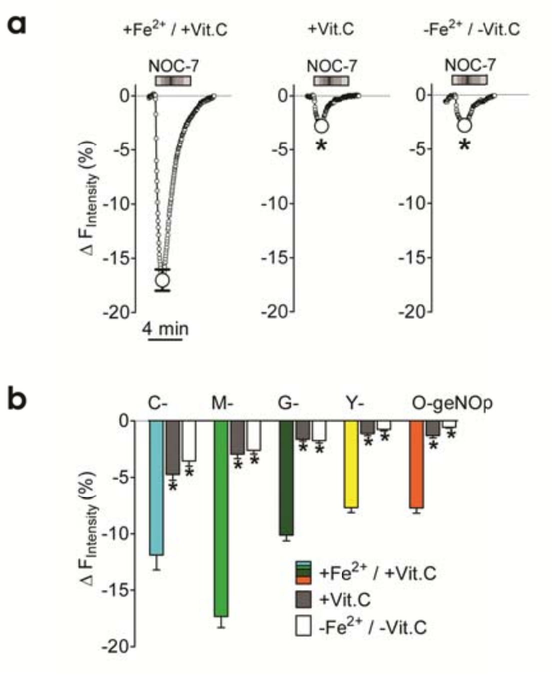

(**a**) Representative average curves (mean ± SEM) showing the effects of 10 µM NOC-7 on the fluorescence intensity over time of M-geNOp expressed in HeLa cells. Cells expressing M-geNOp were either pretreated for 10 min. with a buffer containing 1 mM iron(II) fumarate and 1 mM vitamin C (+Fe^{2+} / +Vit.C, left panel n=12) or 1 mM vitamin C alone (+Vit.C, middle panel n=11) or without any pretreatment (-Fe^{2+} / -Vit.C, right panel n=7). *$P < 0.05$ vs. +Fe^{2+} / +Vit.C. (**b**) Bars showing maximal fluorescence quenching effects of 10 µM NOC-7 on the differently colored geNOps that were expressed in HeLa cells. As shown in panel a cells were either pretreated for 10 min. with a buffer containing 1 mM iron(II) fumarate and 1 mM vitamin C (+Fe^{2+} / +Vit.C, colored bars; n=10 for C-geNOp; n=12 for M-geNOp; n=9 for G-geNOp; n=13 for Y-geNOp; n=9 for O-geNOp) or 1 mM vitamin C alone (+Vit.C, grey columns; n=9 for C-geNOp; n=11 for M-geNOp; n=12 for G-geNOp; n=16 for Y-geNOp; n=5 for O-geNOp) or without any pretreatment (-Fe^{2+} / -Vit.C, white columns; n=7 for C-geNOp; n=7 for M-geNOp; n=12 for G-geNOp; n=14 for Y-geNOp; n=9 for O-geNOp). mean ± SEM. *$P < 0.05$ vs. +Fe^{2+} / +Vit.C.

Supplementary Figure 7 I Generation of a non-functional geNOp by disrupting the non-heme iron binding pocket

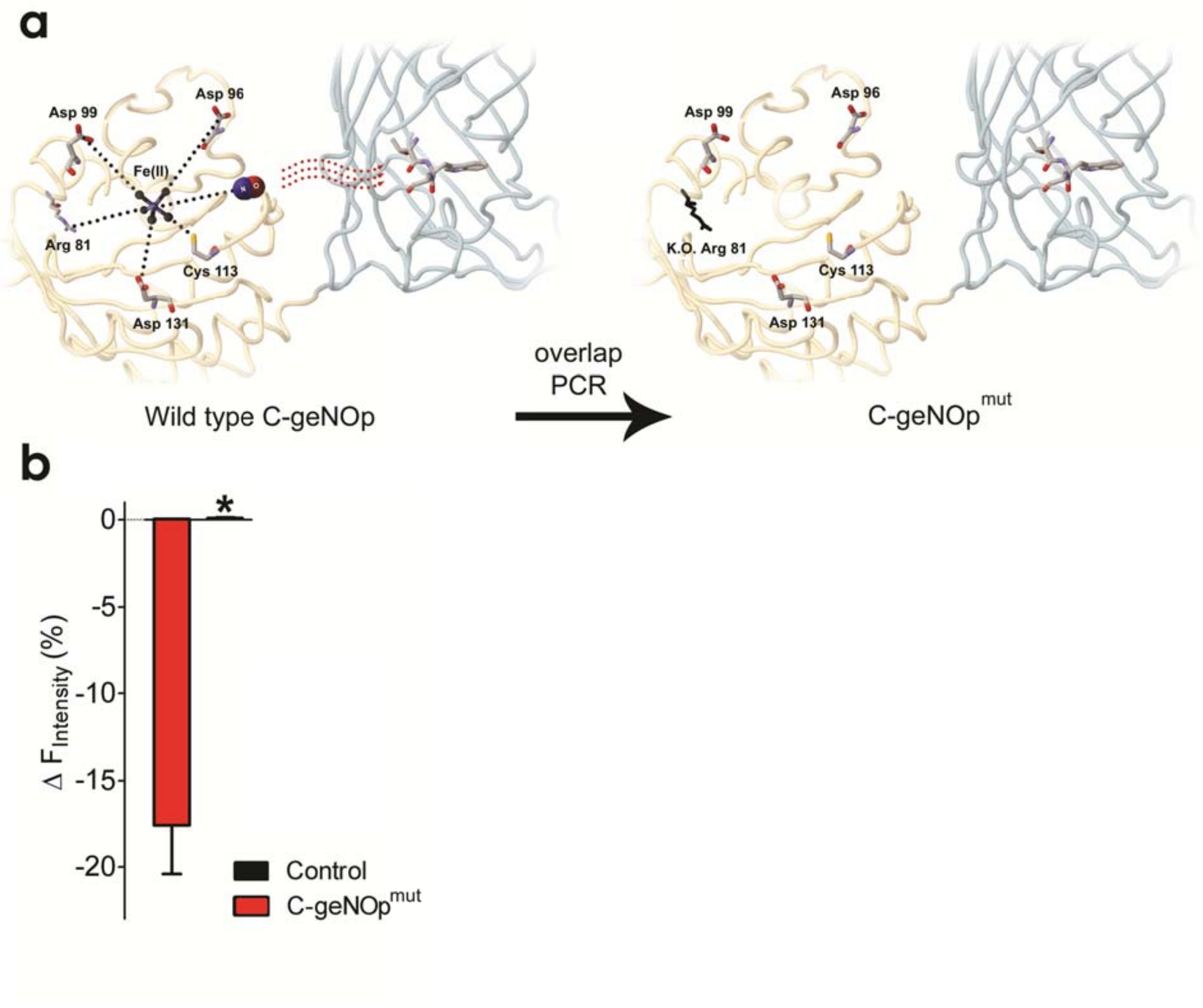

(**a**) Schematic illustration of the generation of C-geNOp^{mut} from wild type C-geNOp. C-geNOp^{mut} is due to the lack of R81 unable to incorporate iron (II) and, hence, bind NO•. (**b**) Bars represent maximal responses of control cells (cells expressing wild-type C-geNOp, red column, n=5) and cells expressing C-geNOp$^{mut.}$ (black column, n=6). mean ± SEM in response to 10 µM NOC-7. HeLa cells were used for these experiments. *P < 0.05 vs. Control.

Supplementary Figure 8 I NO• does not affect fluorescence of FPs that are not fused to the GAF domain

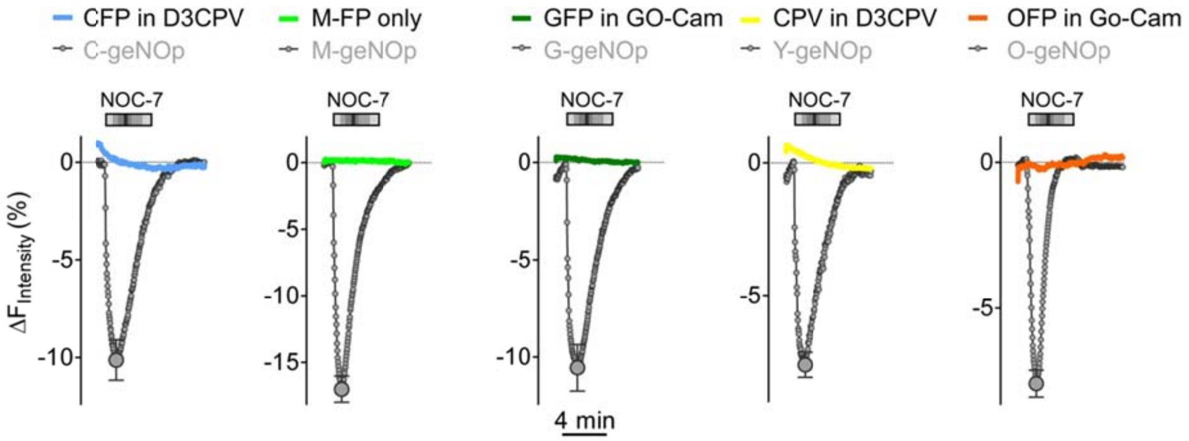

Curves represent average fluorescence intensities (mean ± SEM) over time in response to 10 µM NOC-7. The fluorescent constructs were expressed in HeLa cells, which were stored in a buffer containing 1 mM iron(II) fumarate and 1 mM vitamin C for 10 minutes prior to microscopy. Grey curves are control experiments as shown in Fig. 1c. C-geNOp (n=10) were compared with CFP as component of the CFP/YFP-based cameleon D3CPV (cyan curve, n=8). M-geNOp (n=12) containing the green emitting fluorescent protein (GEM) was compared with the respective GEM expressed in HeLa cells (light green curve, n=16). The GFP containing G-geNOp (n=11) was compared with GFP fluorescence from the red-shifted cameleon GO-Cam (dark green curve, n=7). Y-geNOp containing the circularly permuted citrine (CPV) (n=13) was compared with CPV as component of the cameleon D3CPV (yellow curve, n=11). O-geNOp based on the monomer Kusabira Orange fluorescent protein (OFP) (n=9) was compared with OFP expressed as the red-shifted cytosolic cameleon GO-Cam (orange curve, n=7).

Supplementary Figure 9 I In contrast to Hyper G-geNOp does not respond to H_2O_2

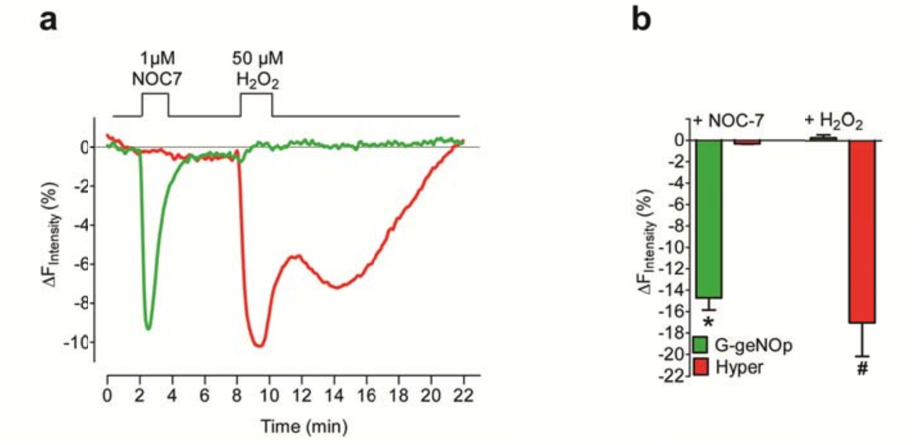

(**a**) The green curve represents fluorescence of G-geNOp over time in response to 1 µM NOC-7 and 50 µM H_2O_2. The red curve shows fluorescence over time of mitochondria targeted Hyper under the same conditions. (**b**) Statistics of average maximal fluorescence changes of G-geNOp (left green column, n=6) and mitochondria targeted Hyper (left red column, n=7) in response to 1 µM NOC-7 (left columns) and in response to 50 µM H_2O_2 (G-geNOp, green right column, n=8; mitochondria targeted Hyper, red right column, n=10) using HeLa cells. *$P < 0.05$ vs. 50 µM H_2O_2; # $P < 0.05$ vs. 1 µM NOC-7.

Supplementary Figure 10 I Selectivity tests using the GPI-anchored C-geNOp (GPI-C-geNOp)

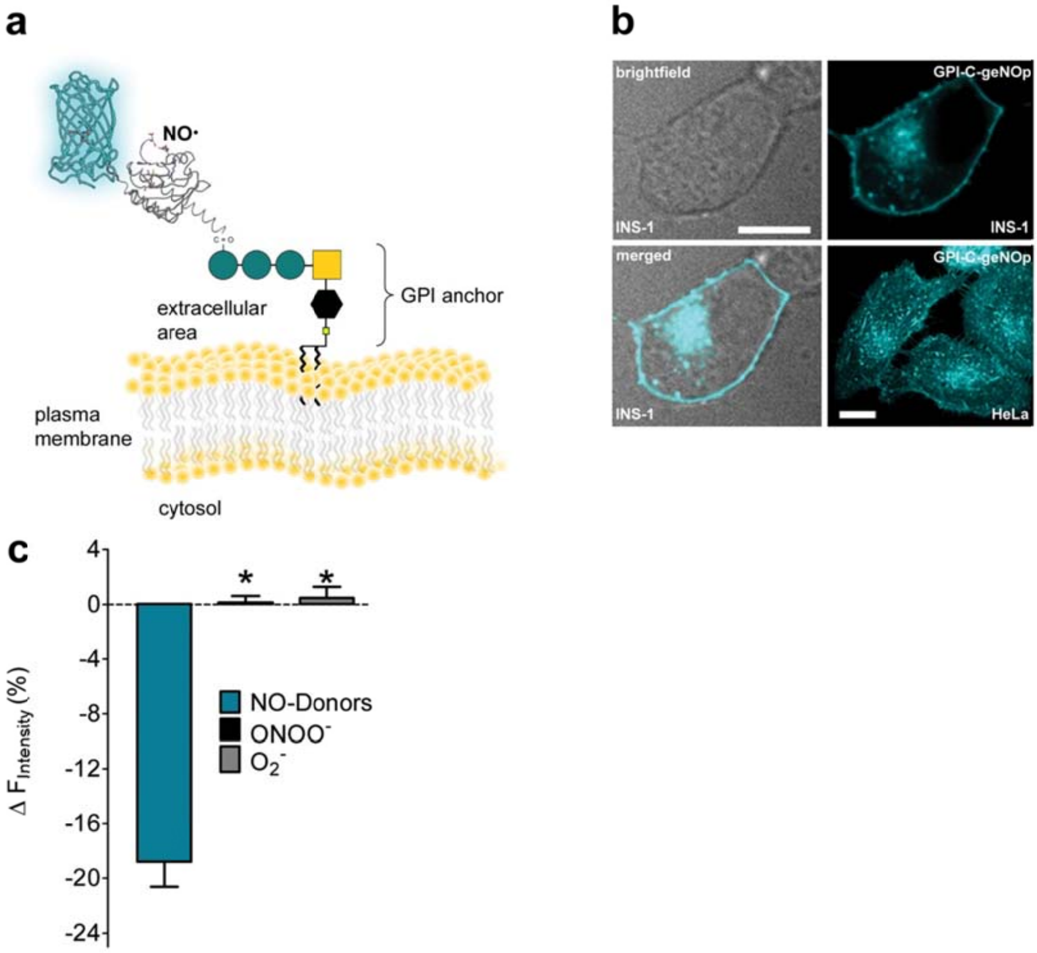

(**a**) Schematic illustration of GPI-C-geNOp (**b**) Representative confocal images of cells (INS-1, upper images and left lower image; and HeLa cells in the lower right image) expressing GPI-C-geNOp. White bar represents 10 µm. (**c**) Columns showing maximal fluorescence changes ± SEM of GPI-C-geNOp at the plasma membrane (region of interest were selected at the periphery of cells) in response to 10 µM NOC-7 and 5 mM SNP (NO-Donors; blue column, n= 19), 10 µM peroxynitrite ($ONOO^-$; black column, n=6), and 100 µM potassium superoxide ($O2^-$; grey column, n=8). HeLa cells expressing GPI-C-geNOp were treated for 3-4 minutes with the radicals using a perfusion system. *$P < 0.05$ vs. NO-Donors.

Supplementary Figure 11 I The functionality of G-geNOp is preserved over a wide pH range

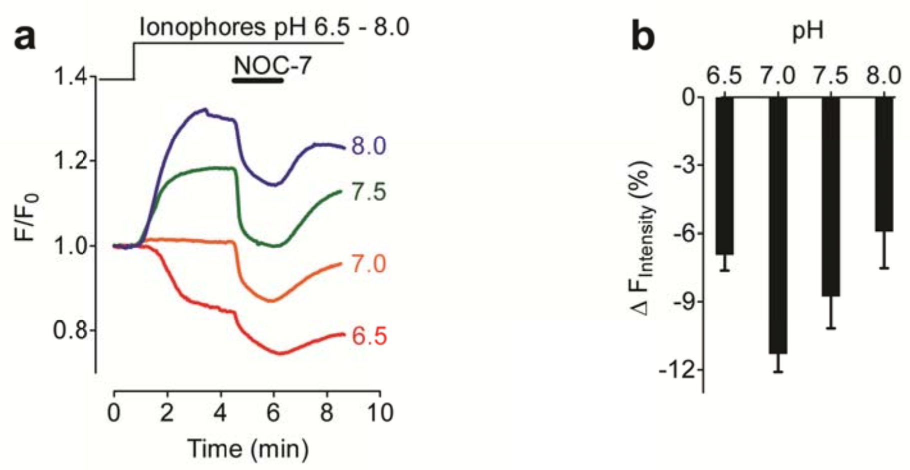

(**a**) Representative curves showing the effects of 10 µM NOC-7 on the fluorescence intensity over time of G-geNOp expressed in HeLa cells at various pH values: pH 6.5 (red curve, n=13); pH 7.0 (orange curve, n=10); pH 7.5 (green curve, n=7); pH 8.0 (blue curve, n=10). Cells expressing G-geNOp were treated with a mixture of nigericin, monensin and ionomycin in order to clamp the intracellular pH as described in methods. (**b**) Bars showing maximal fluorescence quenching effects of 10 µM NOC-7 at the different pH values as indicated in panel a.

Supplementary Figure 12 I pH sensitivity of differently colored geNOps in cells

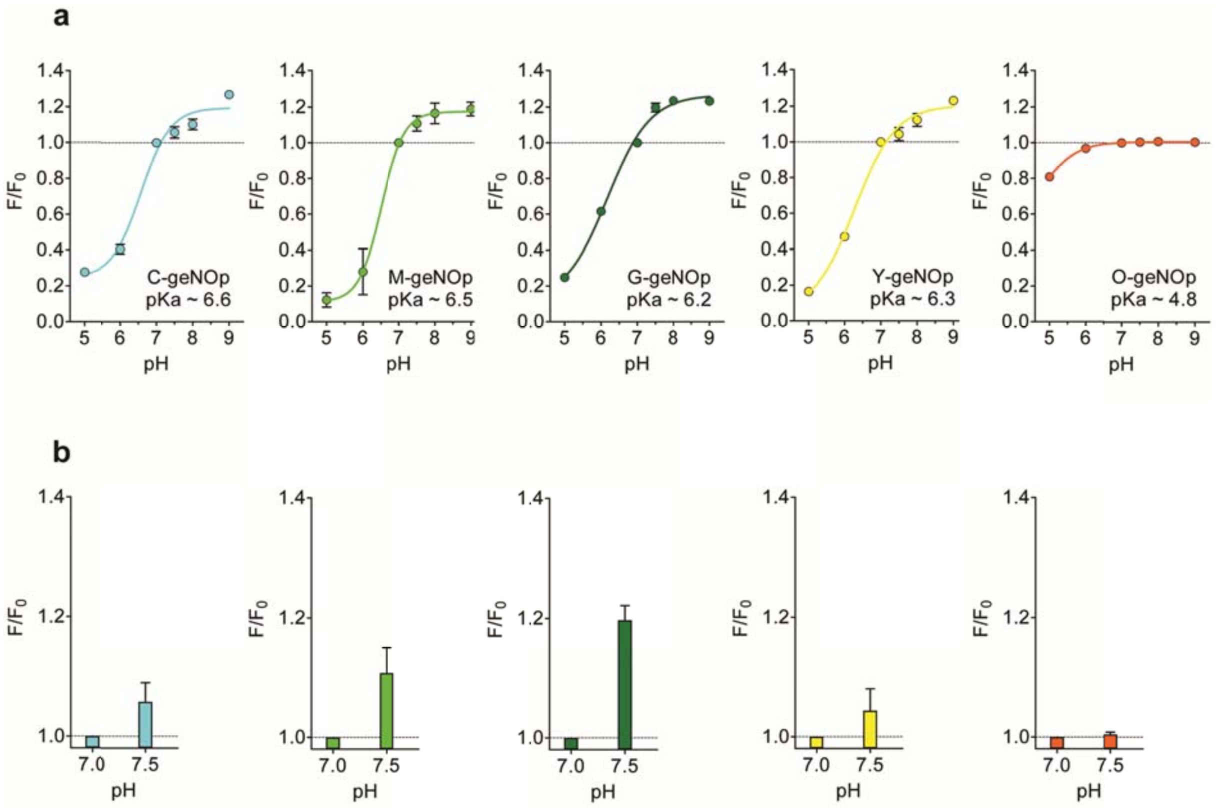

(**a**) pH titration curves of the differently colored geNOps (C-geNOp, n= 6; M-geNOp, n=3; G-geNOp, n=6, Y-geNOp, n=9; O-geNOp, n=5). Cells expressing geNOps were treated with a mixture of monensin, nigericin, ionomycin, and FCCP to fully permeabilize cells for protons. Values were normalized by calculating F/F_0, whereas the fluorescence intensity at pH 7.0 was defined as F_0. (b) Columns represent fluorescence fold change between pH 7 and 7.5 of the differently colored geNOps. Data are extracted from the curves shown in panel **a**.

Supplementary Figure 13 I The NO• insensitive mutated geNOps (geNOp^{mut}) keep their pH sensitivity

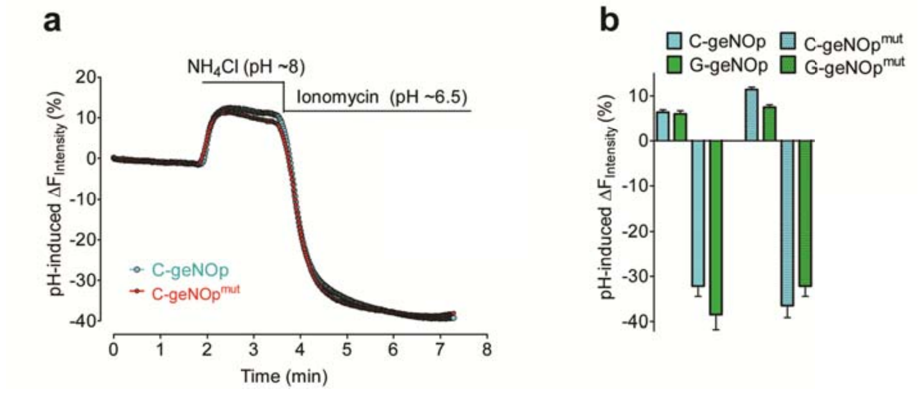

(**a**) Curves showing fluorescence of single HeLa cells over time expressing C-geNOp (left panel) or C-geNOp^{mut} (right panel) in response to 3 mM ammonium chloride (NH_4Cl), which alkalize the intracellular milieu, and 3 µM ionomycin at a pH of 6.5 for an efficient acidification of cells. (**b**) Columns represent maximal average values of fluorescence changes upon alkalization (positive values) and acidification (negative values) of C-geNOp (left, cyan columns, n=14), G-geNOp (left dark green columns, n=14), C-geNOp^{mut} (right, cyan columns=12), and G-geNOp^{mut} (right, dark green columns, n=15).

Supplementary Figure 14 I Mitochondria targeted mtC-geNOps and mtG-geNOp show clear organelle localization

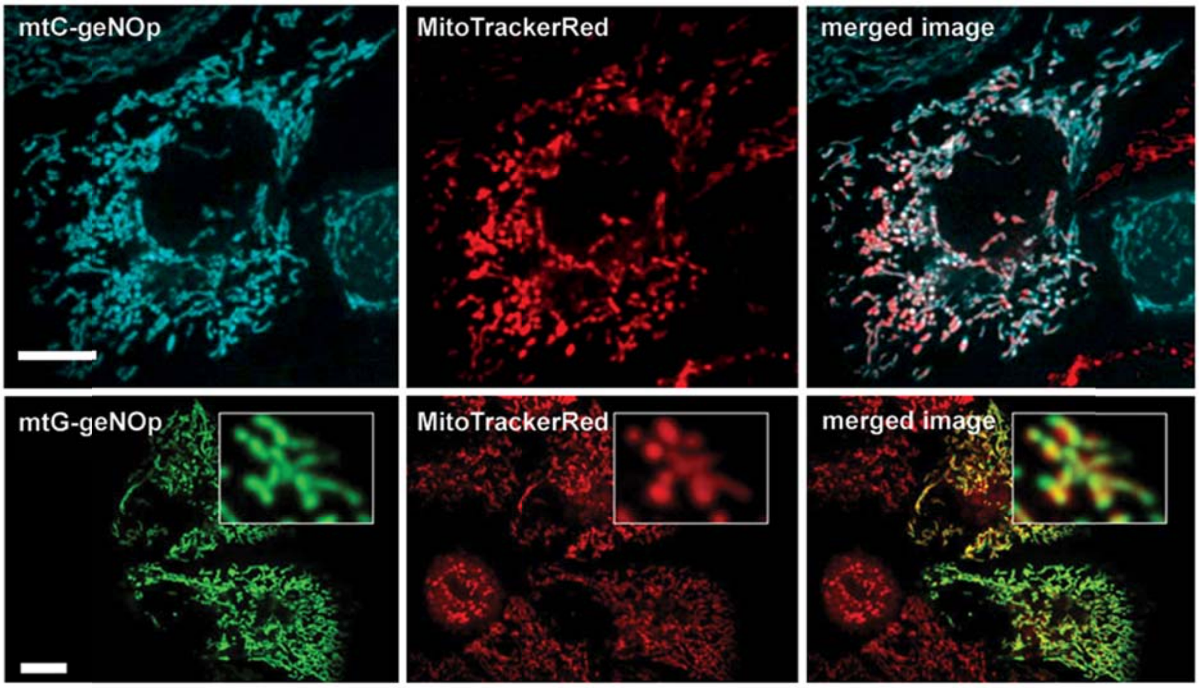

Upper images: Representative confocal images of HeLa cells expressing mtC-geNOp (left image). Cells were also loaded with MitoTrackerRed® (middle panel), which colocalized with the mtC-geNOp fluorescence (right image). Lower Images: HeLa cells expressing G-geNOp (left image) were loaded with MitoTrackerRed® (middle panel), which colocalized with the mtG-geNOp fluorescence (right image). Deviations in colocalization are due to movements of the organelles. Both bars represent 10 µm. Images were taken using a array confocal laser scanning microscope using a 100 x objective.

Supplementary Figure 15 I Imaging of cellular NO• signals with C-geNOp unveils different NO•-releasing kinetics and capacities of various NO• donors

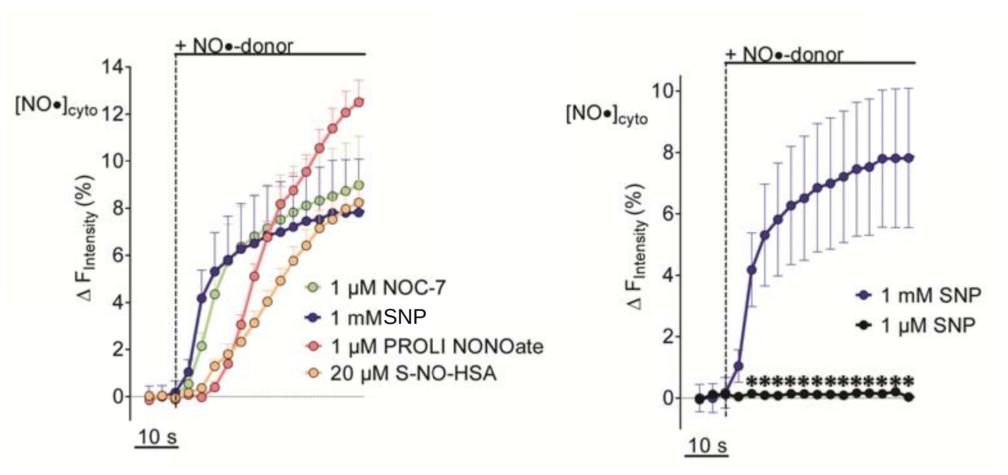

Average curves over time representing cellular NO• increases of HeLa upon addition of different NO• donors. Cells expressing C-geNOp were treated either with 1 µM NOC-7 (left panel, green curve, n=10) or 1 mM SNP (both panels, blue curves, n=7) or 1 µM PROLI NONOate (left panel, red curve, n=16) or 20 µM S-NO-HSA (left panel, orange curve, n=6). 1 µM SNP was ineffective to elevate cellular NO• levels (right panel, black curve, n=10). *$P < 0.05$ vs. 1 mM SNP.

Supplementary Figure 16 I Measurements of S-NO-HSA-released NO• with a porphyrinic nanosensor and geNOps

a

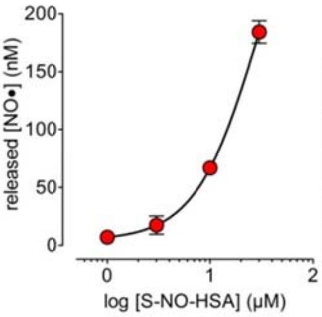

S-NO-HSA (µM)	[NO•] (nM)
1	7.09 ± 3.4
3	17.40 ± 7.9
10	66.80 ± 4.8
30	184.30 ± 9.7

b

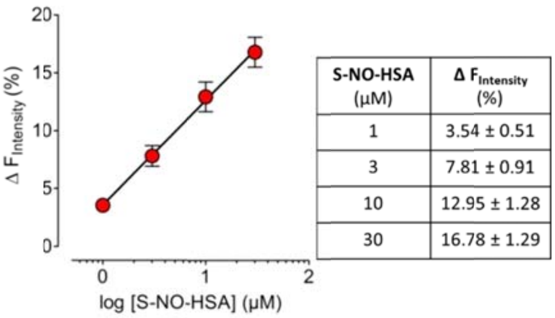

S-NO-HSA (µM)	$\Delta F_{Intensity}$ (%)
1	3.54 ± 0.51
3	7.81 ± 0.91
10	12.95 ± 1.28
30	16.78 ± 1.29

a) Semi-logarithmic XY-plot showing the amount of NO• released (in nM, y-axis) by different S-NO-HSA concentrations. NO• released by 1 µM, 3 µM, 10 µM, and 30 µM S-NO-HSA (mean ±SD, n=3 for all concentrations) was determined using a porphyrinic nanosensor as described in methods. (**b**) Semi-logarithmic XY-plot showing fluorescence quenching ($\Delta F_{Intensity}$ in %, Y-axis) of C-geNOp expressed in HeLa cells in response to 1 µM, 3 µM, 10 µM, and 30 µM S-NO-HSA (mean ±SEM, n=5 for all concentrations).

Supplementary Figure 17 I Manipulation of cellular NO• levels in primary cardiomyocytes using NO• donors

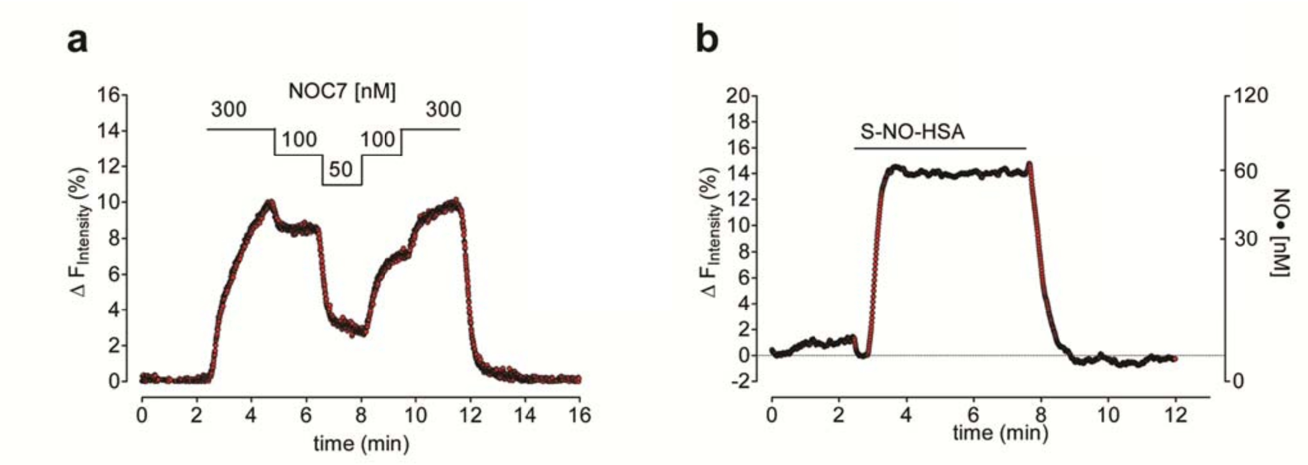

(**a**) Representative cellular NO• dynamics that were evoked by addition of different NOC-7 concentrations in a single cardiomyocyte expressing G-geNOp. (**b**) Stable NO• elevation induced by 10 µM S-NO-HSA in a single cardiomycyte expressing G-geNOp.

Supplementary Figure 18 I C-geNOp^{mut} does not respond upon Ca^{2+} mobilization with histamine in EA.hy926 cells

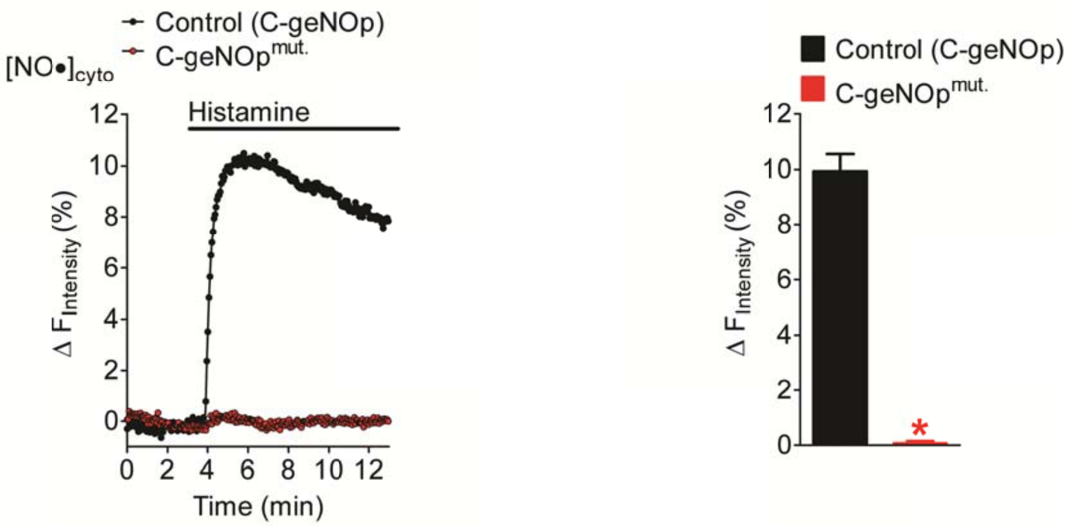

Cytosolic NO• signals in EA.hy926 cells expressing either functional C-geNOp (Control, black curve, black column, n=23) or C-geNOp^{mut} that lacks the essential arginine in position 81 (R81) of the non-heme iron(II) center within the NO• binding GAF domain (red curve and red column, n=8). Cells were treated with 100 µM of the IP_3-generating agonist histamine in the presence of extracellular Ca^{2+} (2mM). Bars represent Δ_{max} mean ± SEM values. *P < 0.05 vs. Control.

Supplementary Figure 19 I Ca^{2+} entry in endothelial cells facilitates cellular NO• production

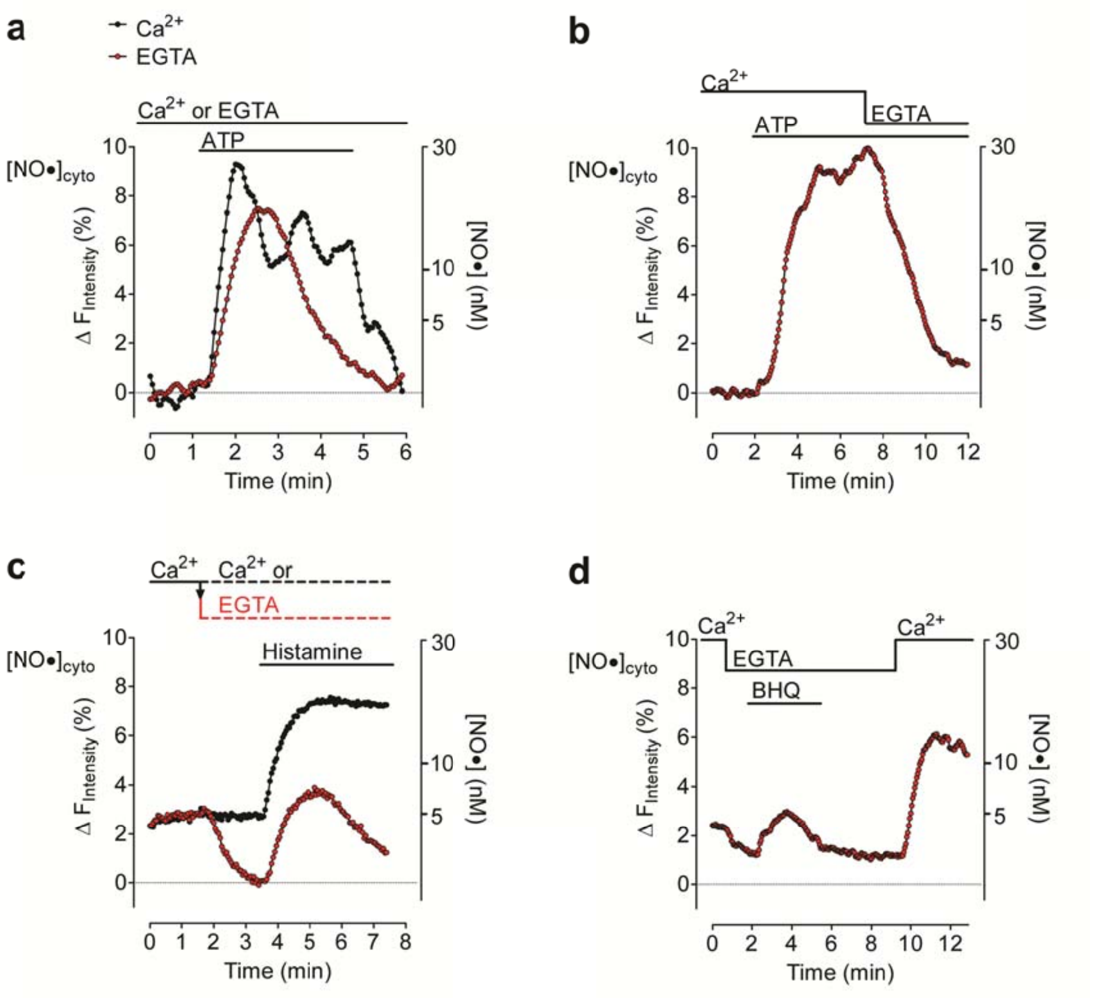

(**a**) Cytosolic NO• signals in EA.hy926 cells expressing C-geNOp. Cells were treated with 100 µM of the IP$_3$-generating agonist ATP in the presence of extracellular Ca^{2+} (black oscillating curve) or in the absence of Ca^{2+} (1 mM EGTA, red transient curve. (**b**) Representative NO• dynamics of a single endothelial cell expressing C-geNOp. The cell was stimulated with 100 µM ATP in the presence of Ca^{2+}; as indicated Ca^{2+} was removed by 1 mM EGTA during cell stimulation using a gravity-based perfusion system. (**c**) EA.hy926 cells were stimulated with 100 µM histamine either in the presence (black curve) or absence (1 mM EGTA, red curve) of Ca^{2+}. (**d**) Single cell (EA.hy926) NO• dynamics upon ER Ca^{2+} mobilization with the SERCA inhibitor BHQ in 1 mM EGTA and the subsequent re-addition of 2 mM Ca^{2+}.

Supplementary Figure 20 I N^G-nitro-L-arginine blocks histamine-induced NO• signals in endothelial cells

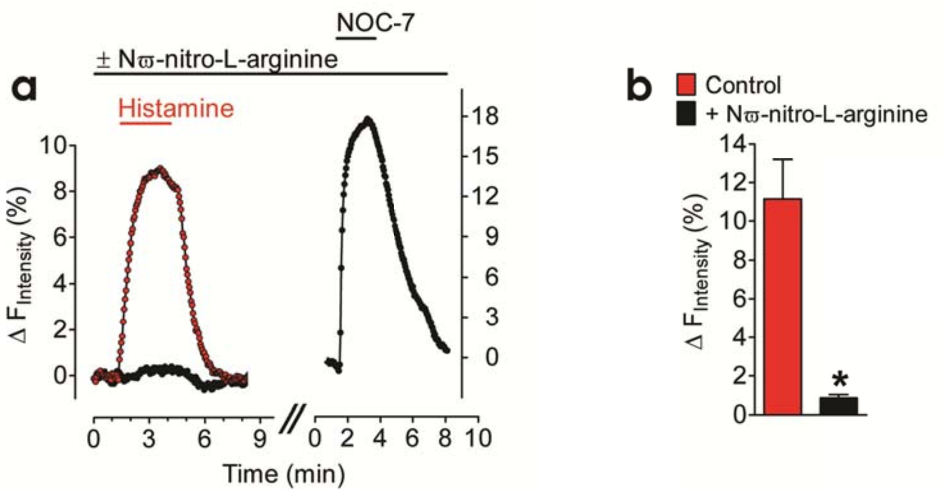

(**a**) Representative NO• signals versus time of endothelial cells expressing C-geNOp (right panel, inverted curves are shown). Cells were treated with 10 µM histamine in the presence of extracellular Ca^{2+} under controlled conditions (red inverted curve) or in the presence of 1 mM N^G-nitro-L-arginine (black inverted curve). The cell that was incubated with N^G-nitro-L-arginine was also treated with 10 µM NOC-7, as indicated. (**b**) Columns represent maximal geNOps signals as shown in panel a. Control conditions (red column, n=5) and in the presence of the NOS inhibitor (black column, n=10). *P < 0.05 vs. Control.

Supplementary Figure 21 I Co-imaging of cytosolic Ca^{2+} and NO• signals in a single endothelial cell

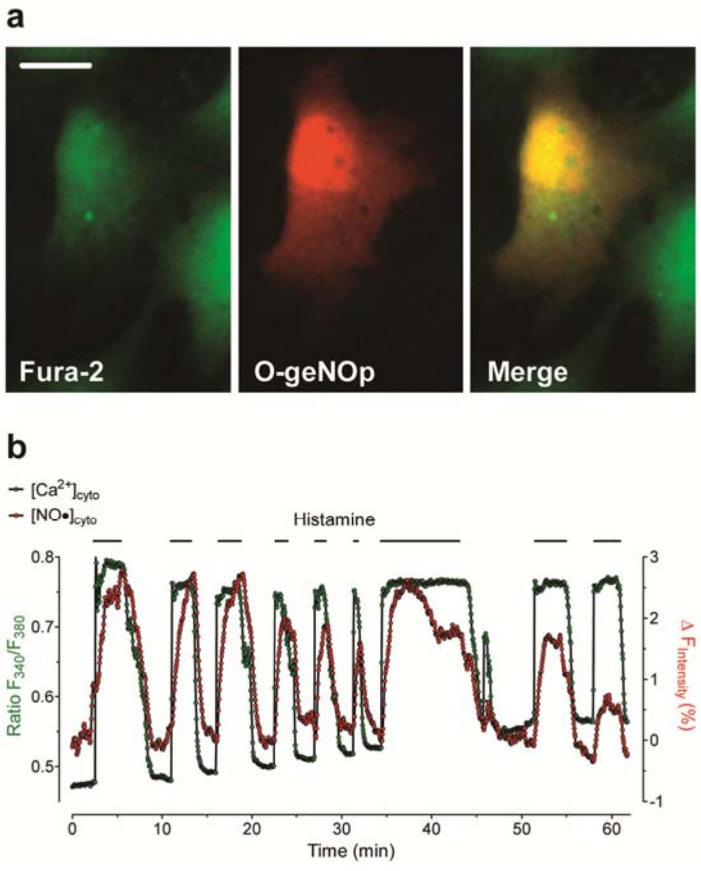

(**a**) Representative wide field fluorescence images of an EA.hy926 cell expressing O-geNOp (middle image) additionally loaded with fura-2/am (left image) and the respective overlay (right image). The scale bar refers to 20 µm. (**b**) Curves represent simultaneous recordings of cellular Ca^{2+} (dark green ratio curve) and NO• (red inverted curve) signals over time of a single fura-2/am-loaded endothelial cell expressing O-geNOp as shown in panel (a). The cell was stimulated repetitively with 100 µM histamine for different time periods as indicated. Cells were kept in a physiological buffer solution containing 2mM Ca^{2+}.

Supplementary Figure 22 I Elimination of the spectral crosstalk between (mt)C-geNOp and (mt)G-geNOp using spectral unmixing

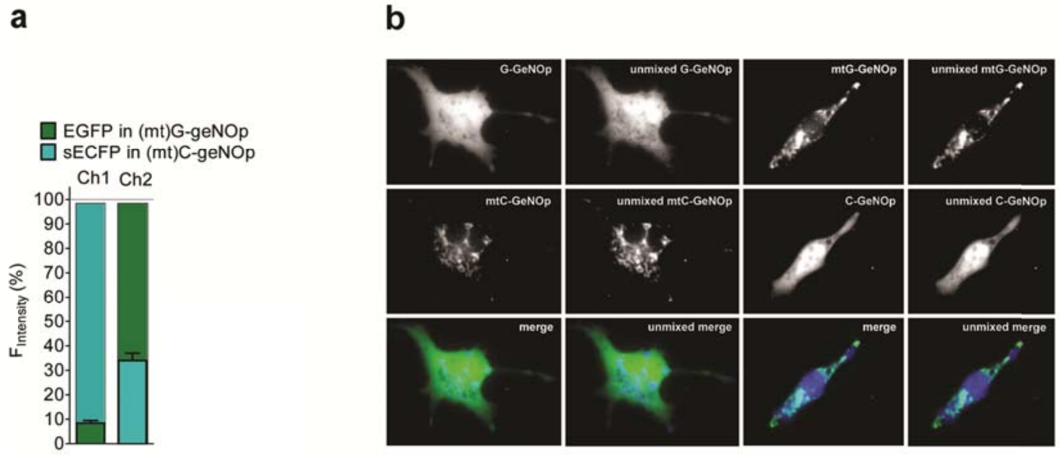

(**a**) Columns represent average fluorescence intensities of (mt)G-geNOp in channel 1(Ch1, optimized for sECFP fluorescence, n=19) and (mt)C-geNOp in channel 2 (Ch2, optimized for EGFP fluorescence, n=12) showing respective spectral crosstalk between the respective FPs (**b**) Images of EA.hy926 cells expressing cytosolic G-geNOp together with mtC-geNOp (left image pairs) prior to (very left image row) and after spectral unmixing (second image row from left). Vice versa mtG-geNOp was expressed together with cytosolic C-geNOp prior to (third image row) and after spectral unmixing (very right image row).

Supplementary Figure 23 I Simultaneous recordings of cytosolic and mitochondrial NO• signals

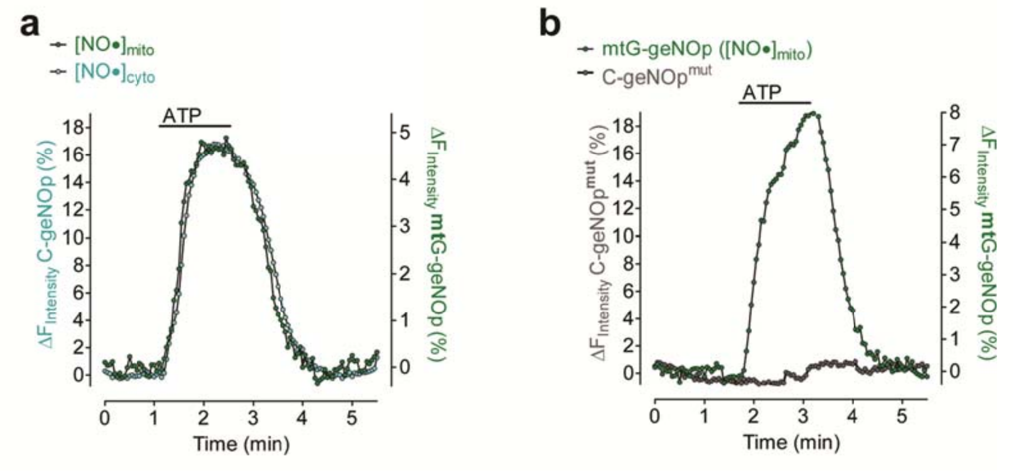

(**a**) Representative simultaneous recordings of mtG-geNOp (green curve) and cytosolic C-geNOp signals (cyan curve) over time in a single EA.hy926 cell in response to 100 µM ATP. (**b**) Respective representative experiment as shown in panel (a) in which C-geNOp^{mut} was used instead of the NO• sensitive probe. (n=4, for all conditions).

Supplementary Table 1 I EC_{50} values of NOC-7 on differently colored geNOps

NO• probe	EC_{50} in nM	**95% Confidence Intervals**	**R^2**
C-geNOp	94.1	77.1 to 115.0	0.9957
M-geNOp	87.2	71.8 to 106.0	0.9968
G-geNOp	50.0	37.0 to 67.6	0.9953
Y-geNOp	70.7	38.8 to 128.8	0.9868
O-geNOp	51.3	40.7 to 64.6	0.9969

Supplementary Note 1

The fact that we found a direct proportionality between the starting fluorescence and the fluorescence decay after NO• binding allows easy relative and absolute quantification by normalization. Since cells with different expression levels of geNOps have been used, this direct proportionality is a key feature of the NO• probes. Otherwise further calibrations on each single geNOp had to be done to compensate for these deviations.

Supplementary Note 2

These data were also used to analyze ka,kd and KD of C-geNOp. To analyze ka,kd and KD kinetics the recordings of consecutive addition and removal of different concentrations of NOC-7 ranging from 1 nM to 100 µM were taken into account (see also Figure 1e and f). After bleach correction of each signal by an exponential fit ([S] = A • e^{Bt} + C) the ΔF was transferred into absolute NO-concentrations using equations for C-geNOp (see Methods). To get the KD-values of C-geNOp the initial speed after addition of each NOC-7 concentrations expressed as the maximal slope was determined. The slope was plotted using a Lineweaver burk diagram to extract KD. By using an exponential decay kinetic ($[NO] = [NO]_0 \bullet e^{-kdt}$) fitted to the wash out step of each Noc-7 concentration kd was determined. Since KD =kd/ka, ka could be calculated. Analysis of kd, KD and ka was done for C-geNOp on three independent experiments with each nine NOC-7 concentrations ranging from 100 µM to 1 nM.

$$\frac{k_d}{k_a} = \frac{[P] \cdot [NO]}{[PNO]} = K_D$$

Association Phase:

$$R_{eq} = \frac{k_a \cdot R_{max} \cdot [NO]}{k_d + k_a \cdot [NO]} = \frac{R_{max} \cdot [NO]}{K_D \cdot [NO]}$$

R(t) is Signal at timepoint t and R(max) is maximal Signal.

Dissociation Phase:

$$R_t = R_{min} + R_0 \cdot e^{-k_d \cdot t}$$

R(t) have to be directly proportional to $[NO]$

	C-geNOp	
	mean	SEM
**KD**	55.227067	4.463026
**kd**	0.040950	0.003642
**ka**	0.000752	0.000092

Supplementary Note 3

Spectral unmixing was performed using the following equations:

$$Fl1 = \frac{B2 \bullet CHI - B1 \bullet CHII}{A1 \bullet B2 - B1 \bullet A2}$$

$$Fl2 = \frac{A1 \bullet CHII - A2 \bullet CHI}{A1 \bullet B2 - B1 \bullet A2}$$

Fl1: is the unmixed (pure) fluorescence of e.g. (mt)C-geNOp in channel 1

Fl2: is the unmixed (pure) fluorescence of e.g. (mt)G-geNOp in channel 2

A1: is the average fluorescence of (pure) fluorophore1 (e.g. (mt)C-geNOp = 0.660) in channel 1

A2: is the average fluorescence of (pure) fluorophore1 (e.g. (mt)C-geNOp = 0.380) in channel 2

B1: is the average fluorescence of (pure) fluorophore2 (e.g. (mt)G-geNOp = 0.084) in channel 1

B2: is the average fluorescence of (pure) fluorophore2 (e.g. (mt)G-geNOp = 0.916) in channel 2

CHI: is the mixed fluorescence signal (image) in channel 1

CHII: is the mixed fluorescence signal (image) in channel 2
